# *Trp53* deficient mice predisposed to preterm birth display region-specific lipid alterations at the embryo implantation site

**DOI:** 10.1038/srep33023

**Published:** 2016-09-13

**Authors:** Ingela Lanekoff, Jeeyeon Cha, Jennifer E. Kyle, Sudhansu K. Dey, Julia Laskin, Kristin E. Burnum-Johnson

**Affiliations:** 1 Physical Sciences Division, Pacific Northwest National Laboratory, Richland, WA, 99352, USA; 2 Department of Chemistry-BMC, Uppsala University, Sweden; 3 Division of Reproductive Sciences, Cincinnati Children’s Hospital Medical Center, Cincinnati, OH 45229, USA; 4 Biological Sciences Division, Pacific Northwest National Laboratory, Richland, WA, 99352, USA.

## Abstract

Here we demonstrate that conditional deletion of mouse uterine *Trp53 (p53*^*d/d*^), molecularly linked to mTORC1 activation and causally linked to premature uterine senescence and preterm birth, results in aberrant lipid signatures within the heterogeneous cell types of embryo implantation sites on day 8 of pregnancy. *In situ* nanospray desorption electrospray ionization mass spectrometry imaging (nano-DESI MSI) was used to characterize the molecular speciation of free fatty acids, monoacylglycerol species, unmodified and oxidized phosphatidylcholine (PC/Ox-PC), and diacylglycerol (DG) species within implantation sites of *p53*^*d/d*^ mice and floxed littermates. Implantation sites from *p53*^*d/d*^ mice exhibited distinct spatially resolved changes demonstrating accumulation of DG species, depletion of Ox-PC species, and increase in species with more unsaturated acyl chains, including arachidonic and docosahexaenoic acid. Understanding abnormal changes in the abundance and localization of individual lipid species early in the progression to premature birth is an important step toward discovering novel targets for treatments and diagnosis.

Preterm birth and prematurity are global issues with immediate and long-term social and economic consequences. Thirteen million premature births and more than three million stillbirths occur each year. Prematurity is a direct cause of 35% of all neonatal deaths annually, totaling more than one million deaths worldwide^1^. Furthermore, many babies who survive premature birth suffer from serious long-term disabilities such as neurodevelopmental impairments, behavioral problems, and respiratory illnesses^2^. Mice with conditional deletion of uterine *Trp53 (p53*^*d/d*^) serve as a powerful model to study the molecular landscape associated with spontaneous preterm labor^3^,^4^,^5^. Whereas all floxed (*p53*^*f/f*^) mice experience labor between days 19 and 20 of pregnancy with full complement of offspring, more than 50% of *p53*^*d/d*^ mice lacking uterine p53 deliver preterm on days 17 or 18 and lose 100% of the offspring through stillbirth or neonatal death^3^. We have previously shown that early aberrations during decidualization (e.g., premature decidual senescence) can result in adverse pregnancy outcomes, such as preterm birth^3^. The process of decidualization is maximal on day 8 of pregnancy to accommodate and support the developing embryo before establishment of a functional placenta, which forms at the mesometrial (M) pole of the uterus. Mice with conditional uterine deletion of p53 have premature decidual growth restriction with polyploidy, accelerated terminal differentiation and decidual senescence on day 8 of pregnancy^3^. We previously reported markedly enhanced levels of cyclooxygenase 2 (COX-2), prostaglandin F synthase (PGFS) and prostaglandin F_2α_ (PGF_2α_) on day 16 of pregnancy^3^. Prostaglandin (PG) species are converted from arachidonic acid, a free fatty acid originating from the metabolism of a variety of lipid species by cyclooxygenases (COX). Comparable signatures of decidual senescence with increased COX signaling were observed in women undergoing preterm birth, making lipid metabolism and signaling clinically relevant^5^.

The tumor suppressor p53 maintains genomic stability by triggering cell cycle arrest, DNA repair or apoptosis in response to cellular stresses such as DNA damage, in addition to broader cellular functions. Further, p53 modulates lipid metabolism^6^ and COX signaling is increased in mouse uterine tissue deficient of p53 expression^3^. We have previously reported proteomic comparisons of decidua from *p53*^*f/f*^ and *p53*^*d/d*^ implantation sites on day 8, revealing that *Trp53* deficiency negatively affects antioxidant status and ATP production due to mitochondrial dysfunction^7^. Additionally, we showed a decrease of enzymes in the β-oxidation pathway, the process by which fatty acids are metabolized in the mitochondria^7^.

To investigate regional lipid alterations of *p53*^*d/d*^ implantation sites on day 8 of pregnancy, we used nanospray desorption electrospray ionization mass spectrometry imaging (nano-DESI MSI)^8^,^9^,^10^. We have previously used nano-DESI MSI for three-dimensional and MS/MS imaging of lipids and metabolites in mouse embryo implantation sites on day 6 of pregnancy^11^,^12^. The data acquired with nano-DESI MSI contains information about hundreds of molecules detected at each predefined x and y coordinate across the sample. Consequently, any detected molecule can be visualized as an ion image, depicting its distribution and relative abundance on the tissue section. Moreover, nano-DESI MSI enables quantification and generation of ion images free of matrix effects by use of internal standards to the extraction solvent^13^,^14^,^15^,^16^.

Herein, we employed nano-DESI MSI for examining molecular signatures of preterm birth by comparing the localization and abundance of lipids and lipid metabolites in uterine tissue sections of *p53*^*f/f*^ and *p53*^*d/d*^ mice. We report significant cell-type specific differences in the abundance of diacylglycerol (DG) and oxidized phosphatidylcholine (Ox-PC) species. The significant alterations in DG and Ox-PC abundances between control and p53 knockout mice indicate that *Trp53* deficiency is associated with a severely altered lipid metabolism at an early stage of pregnancy.

## Results

Lipid distributions in embryo implantation sites of mice on day 8 of pregnancy were characterized using nano-DESI MSI. At this early stage of pregnancy decidual cell growth and terminal cell differentiation are maximal. Lipid alterations at this sensitive stage of pregnancy can lead to suboptimal pregnancy outcomes^3^,^17^. The two most distinguishable microenvironments of the implantation site encompass the decidual cells at the AM-pole, where the embryo first implants, and the M-pole, the site of placental development. The decidual cells in the AM-pole will eventually undergo apoptosis to leave room for the growing embryo while the cells in the M-pole will develop into the placenta as the embryo requires nutrition to grow.

### Acyl chain composition is significant for Phosphatidylcholine (PC) localization in implantation sites

**Figure 1:**
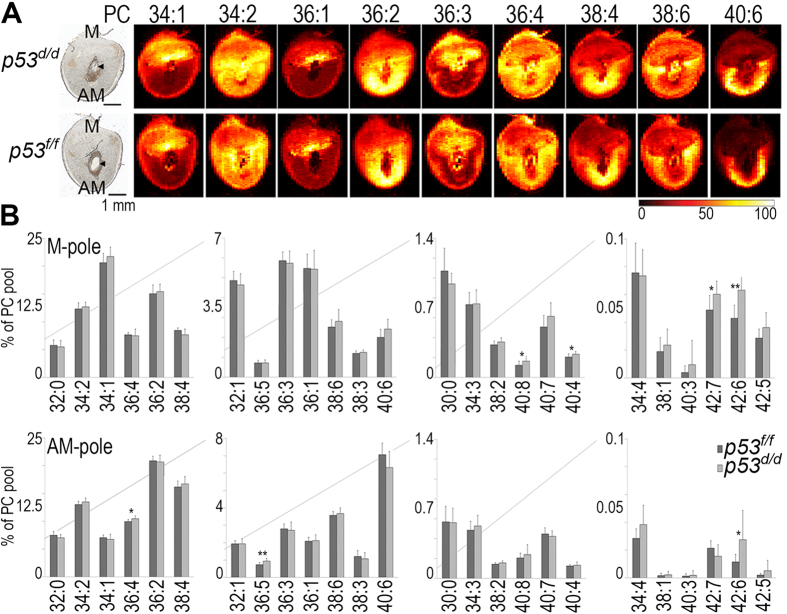
Distributions of PC in mouse embryo implantation sites. (**A**) Optical image and ion images depicting the abundance and localization of abundant PC species in *p53*^*f/f*^ and *p53*^*d/d*^ implantation sites on day 8 of pregnancy; M, uterine mesometrial pole; AM, uterine anti-mesometrial pole; PC, phosphatidylcholine; the embryo is located in the center of the implantation sites; myometrium (muscle layer) encircles the uterus. The intensity scale bar dark to bright represents low to high intensity. Note that the ion images have individual intensity bars ranging between 0–100%, and, therefore, the intensity colors cannot be compared between two images. Scale bar on optical images shows 1 mm. (**B**) The relative abundance of PC species within the total PC pool localized to the M-pole (top row) or the AM-pole (bottom row) in the *p53*^*f/f*^ (dark gray) and *p53*^*d/d*^ (light gray) implantation sites. Significance calculated using the Student’s t-test is depicted with * for p-values < 0.05 and with ** for p-values < 0.01. Error bars show standard deviations for all analyzed tissue sections, 11 for *p53*^*f/f*^ and 10 for *p53*^*d/d*^. PC abbreviations show the total number of acyl chain carbons: total number of double bonds. Non-normalized data and potassium adducts are depicted. Arrowheads denote embryos. Table S2 contains the significance, average values, and standard deviations for these measurements.

Figure 1A shows ion images of nine abundant phosphatidylcholine (PC) species, acquired by nano-DESI MSI. The 12-μm thick tissue sections from the central part of the *p53*^*d/d*^ (top row) and *p53*^*f/f*^ (bottom row) implantation sites containing the embryo were collected on day 8 of pregnancy. The color intensity of the ion images depicts the localization of different PC species to distinct cellular regions of each implantation site^18^. The localizations are highly specific, the difference of a single double bond can restrict the localization of a species to either the mesometrial (M-pole) or antimesometrial (AM-pole) hemisphere of the uterus. For example, PC 36:3 (18:1–18:2) is primarily localized to the M-pole while PC 36:2 (18:0–18:2), with one less double bond, is predominantly localized to the opposite hemisphere of the implantation site, the AM-pole (Fig. 1A). PC 36:1 (18:0–18:1), having only one double bond, is also localized to the M-pole. This distinct distribution of PC species, all of which contain 36 carbons and only differ by having one, two or three double bonds, exemplifies how extremely small variations in lipid structure influence the species-specific localization to unique microenvironments in the tissue. The cellular regions of the AM-pole and the M-pole constitute small subregions of the implantation site tissue sections which only measure ~6 × 4 mm in this study; bulk analysis techniques requiring homogenization would obscure detailed information on the spatial localization of individual lipid species across this complex tissue composed of multiple distinct cell types. However, by use of nano-DESI MSI, regions can be defined post analysis to extract data from specific cellular regions of interest (ROIs) without increasing complexity and uncertainty of the analysis.

Mass spectral abundance data was extracted from the defined regions of the M-pole and AM-pole and statistically compared between mouse implantation sites collected from three p53^*f/f*^ and three *p53*^*d/d*^ mice. The ROI analysis revealed that specific PC species displayed a regional relative change in abundance. In Fig. 1B the abundance of individual PC species within the total PC pool is plotted with the M-pole on top and the AM-pole on bottom for the *p53*^*f/f*^ and *p53*^*d/d*^ implantation sites. PC species with longer and more unsaturated acyl chains exhibited relatively higher abundances within the PC pool of the *p53*^*d/d*^ mice. Specifically, PC 40:8, PC 40:4, PC 42:7, and PC 42:6 are relatively increased in the M-pole and PC 36:4, PC 36:5, and PC 42:6 are relatively increased in the AM-pole of the *p53*^*d/d*^ implantation sites. Interestingly, all of these PC species contain several double bonds, potentially impacting the biophysical properties of the cellular membranes thereby disturbing homeostasis both on the microscale and macroscale level. Microenvironments affected in single cells and across cellular regions of the uterus at day 8 of pregnancy play a role in preterm labor.

### Diacylglycerol (DG) species accumulate in *p53* ^*d/d*^ implantation sites

**Figure 2:**
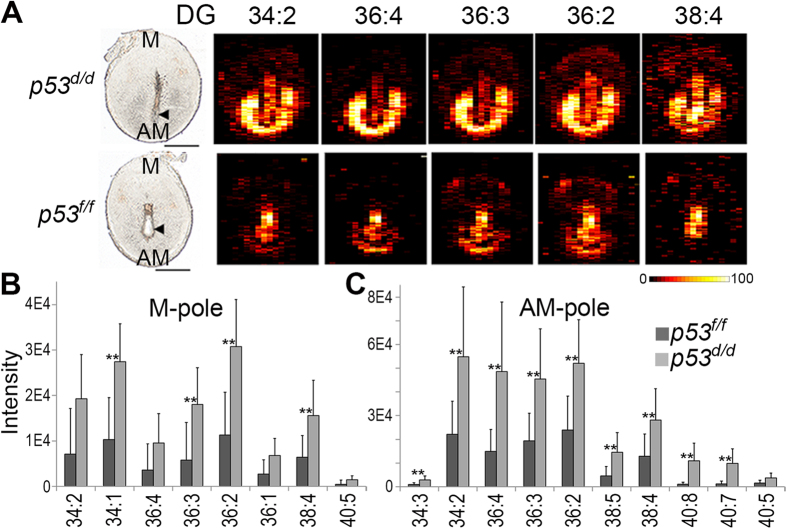
Intensities of specific diacylglycerol (DG) in *p53*^*f/f*^ vs *p53*^*d/d*^ implantation sites on day 8 of pregnancy. All detected DG species are significantly increased (p < 0.05) in the *p53*^*d/d*^ M- and AM-poles, represented as (**A**) nano-DESI images and (**B**,**C**) intensity data. DG abbreviations show the total number of acyl chain carbons: total number of double bonds. The intensity scale bar dark to bright represents low to high intensity. Scale bars show 1 mm. Arrowheads denote embryos. All data p-values < 0.05, **p-values < 0.01. Table S3 contains the significance, average values, and standard deviations for these measurements.

Figure 2A displays ion images of five abundant DG species in implantation sites of *p53*^*f/f*^ (bottom row) and *p53*^*d/d*^(top row) and Fig. 2B,C contain the mass spectral intensity data of several DG species extracted from the defined regions of the M-pole (Fig. 2B) and the AM-pole (Fig. 2C). From Fig. 2B,C it is clear that several DG species significantly accumulate in both the M-pole and the AM-pole of the *p53*^*d/d*^ implantation sites. Figure 2C further shows that DG species in the AM-pole, the site of embryo implantation, have more polyunsaturated fatty acids (PUFAs) in their acyl chains than DG species in the M-pole. DG species play important and diverse roles as both precursors of phospholipids and as second messengers in intracellular communication events. The accumulation of DG will therefore significantly alter cellular events in these regions. Accumulation of neutral lipids including DG has been previously reported after loss of p53 in both mouse embryonic fibroblast cells and mouse liver^6^.

Quantitative polymerase chain reaction (qPCR) was employed to quantify phosphatidic acid phosphatase 2a (PPAP2A) to find pathways of increased DG synthesis. The enzyme PPAP2A converts phosphatidic acid (PA) to DG in an intermediate step for syntheses of PC, phosphatidylethanolamine and triacylglycerol. qPCR was performed on implantation sites from both *p53*^*d/d*^ and *p53*^*f/f*^ mice. The qPCR results (Fig. S12) reveal that PPAP2A is significantly increased (p-value = 0.02; n = 3 females per genotype) in the *p53*^*d/d*^ implantation sites. The previously reported increase in *p53*^*d/d*^ PGF_2α_ signaling through the phosphoinositide signaling system can be linked to DG accumulation, but these qPCR results suggest that DG accumulation in *p53*^*d/d*^ implantation sites is also in part due to increased synthesis of DG in this system by PPAP2A.

### Oxidized phosphatidylcholine (Ox-PC) species are depleted in *p53* ^*d/d*^ implantation sites

**Figure 3:**
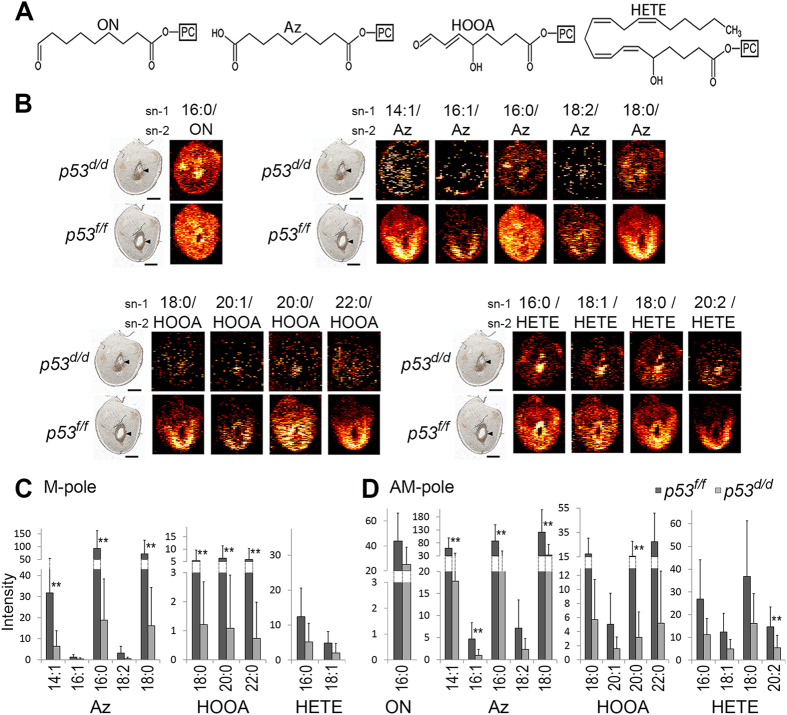
Intensities of selected Ox-PC species in *p53*^*f/f*^ vs *p53*^*d/d*^ implantation sites on day 8 of pregnancy. (**A**) Structures of the sn-2 acyl chain of four groups of Ox-PC species. From left to right; oxononanoyl (ON)-PC, azelayl (Az)-PC, hydroxy-oxooct-enoyl (HOOA)-PC, and hydrox-eicosa-tetra-enoyl (HETE)-PC. (**B**) Ion images of Ox-PC species (the acyl group in the sn-1 position of the PC/the oxidized moiety in the sn-2 position). (**C**) M-pole data. (**D**) AM-pole data. The sn-1 position of each species is depicted where all Ox-PC species are significantly increased (p < 0.05) with ** for p-values < 0.01. Non-normalized data and potassium adducts are depicted. Table S4 contains the significance, average values, and standard deviations for these measurements.

Figure 3A shows the molecular structures of the sn-2 acyl chain in four Ox-PC groups; HOOA-PC (hydroxy-oxooct-enoyl phosphatidylcholine)^19^,^20^; HETE-PC (hydrox-eicosa-tetra-enoyl phosphatidylcholine)^19^; Az-PC (azelayl phosphatidylcholine)^20^; and ON-PC (oxononanoyl phosphatidylcholine)^20^,^21^ which are found in decreased abundance in the *p53*^*d/d*^ implantation sites. The ion images of fourteen individual Ox-PC species, from the above-mentioned groups, are shown in Fig. 3B in *p53*^*f/f*^ and *p53*^*d/d*^ implantation sites. Most Ox-PC species in Fig. 3B are only detected at a few scattered pixels in the *p53*^*d/d*^ tissue, suggesting minimal abundance in the *p53*^*d/d*^ mice. Ox-PC abundances of their littermates are higher and mainly show localization to the site of decidualization in the AM-pole. There are two exceptions: the species ON-PC 16:0 (16:0/ON) and Az-PC 16:0 (16:0/Az) which are more evenly distributed. The plots in Fig. 3 contain the mass spectral intensity data of Ox-PC species from the defined regions of the M-pole (Fig. 3C) and the AM-pole (Fig. 3D) for the *p53*^*f/f*^ and *p53*^*d/d*^ implantation sites. Figure 3C,D show a significantly lower overall abundance of Ox-PC in the cells of *p53*^*d/d*^ uterine tissue, localized both in the M-pole and the AM-pole. Additionally, Fig. 3C,D show that Ox-PC is typically more abundant in the decidual cells of the AM-pole than the M-pole of the uterine sections where they will impact decidualization during embryo development.

In biological systems lower levels of Ox-PC is associated with decreased apoptosis^22^. However, the abundance of caspase 3, a molecular indicator of apoptosis, showed no significant difference between the *p53*^*d/d*^ and the *p53*^*f/f*^ implantation sites (Fig. S13).

### Lipid species with longer more unsaturated acyl chains are relatively higher in the *p53* ^*d/d*^ implantation sites

**Figure 4:**
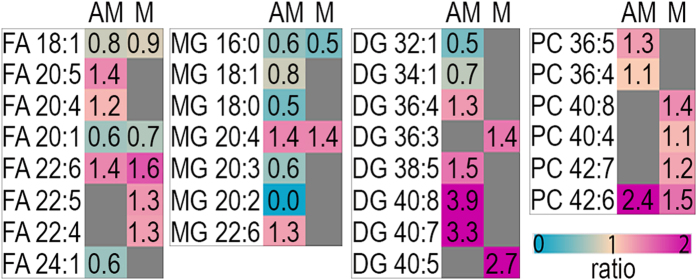
Signal intensity ratio of *p53*^*d/d*^ over *p53*^*f/f*^ for the composition of species within the pool of each molecular class. The pink color show species that are relatively higher in the *p53*^*d/d*^ and the blue color show species that are relatively lower in the *p53*^*d/d*^. DG, Diacylglycerol; FA, Free Fatty Acid; MG, Monoacylglycerol; PC, Phosphatidylcholine; AM, antimesometrial pole; M, mesometrial pole. Lipid abbreviations show the total number of acyl chain carbons: total number of double bonds. Only species which are significantly different (Student’s t-test, p-value < 0.05) are shown. Table S5 contains the significance, average values, and standard deviations for these measurements.

A comparative analysis of the composition of individual species within lipid classes showed that *Trp53* deficiency results in relatively higher abundances of individual lipid species with acyl chains containing PUFAs. Figure 4 shows the relative signal intensity ratios (*p53*^*d/d*^/*p53*^*f/f*^) for individual lipid species in four different lipid classes, free fatty acid (FA), monoacylglycerol (MG), DG, and PC, which are significantly altered in the *p53*^*d/d*^ implantation sites. Lipid species containing 0–1 double bonds (FA 18:1, FA 20:1, FA 24:1, MG 16:0, MG 18:1, MG 18:0, DG 32:1, DG 34:1) are relatively decreased in *p53*^*d/d*^ mice (ratio < 1); whereas, lipid species containing 4–8 double bonds (FA 20:5, FA 20:4, FA 22:6, FA 22:5, FA 22:4, MG 20:4, MG 22:6, DG 36:4, DG 38:5, DG 40:8, DG 40:7, DG 40:5, PC 36:5, PC 36:4, PC 40:8, PC 40:4, PC 42:7, PC 42:6) are relatively increased in *p53*^*d/d*^ mice (ratio > 1). Consequently, species containing more unsaturated acyl chains and longer carbon chains (20–22 carbons for FAs and MGs, 36–40 carbons for DGs, and 36–42 carbons for PCs) are relatively increased in both the AM-pole and M-pole of implantation sites from *p53*^*d/d*^ mice contributing to sever lipid aberrations.

## Discussion

Mice with conditional uterine deletion of *Trp53* exhibit spontaneous preterm birth resulting from abnormal decidualization due to increased premature senescence including increased expression of COX-2, PGFS and PGF_2α_^3^. In this study, we demonstrate that *p53*^*d/d*^ mice additionally exhibit significant and previously uncharacterized aberrations in decidual cell lipid homeostasis. These abnormal lipid profiles detected early in pregnancy (i.e., on day 8 of pregnancy during embryo implantation, the day of maximal decidualization) in *p53*^*d/d*^ mice may be mechanistically important in preterm delivery. Using nano-DESI MSI, we show that day 8 implantation sites from *p53*^*d/d*^ mice have a significant accumulation of multiple DG species and a significant depletion of multiple Ox-PC species in specific cellular regions of the tissue. Furthermore, the *p53*^*d/d*^ mice have a relative accumulation of PUFAs and lipid species containing PUFAs in their acyl chains. The regional lipid profiles characterized for pregnant *p53*^*d/d*^ mice using nano-DESI MSI show previously unknown aberrant lipid signatures, providing novel clues into the molecular mechanism behind preterm birth.

DG is only present at 1 mole% of the lipids in biological membranes, but still plays a profound role in lipid metabolism, membrane function and signaling^23^,^24^,^25^. For example, DG functions as an allosteric activator of protein kinase C in phosphoinositide signaling, a common pathways used by growth factors. Additionally, phosphoinositide signaling controls calcium channels relevant to myometrial contractions^26^. We show that this accumulation of DG in mice with uterine *Trp53* deficiency also results from an overexpression of PPAP2A. Reproductive deficits, resulting from abnormal lipid metabolism, have been reported in PPAP2A over expressing mothers yielding pups with reduced body size and weight^27^. Based on our results we suggest a novel role for DG in early pregnancy correlated to the previously reported increase of PGF_2α_ signaling through the phosphoinositide signaling system^3^.

Previously, we reported the down-regulation of several antioxidant enzymes and proteins involved in mitochondrial ATP production in the *p53*^*d/d*^ uterine tissue and suggested that *Trp53* deficiency lead to mitochondrial dysfunction^7^. Specifically, the mitochondrial β-oxidation pathway is important for decidualization of endometrial stromal cells in both humans and mice^28^. The β-oxidation pathway metabolizes FA to generate energy in the form of FADH2, NADH and acetyl CoA which are important coenzymes in ATP production. During this process, oxygen (O_2_) is converted into water, however, 1–2% escapes this reduction to instead form Reactive Oxygen Species (ROS)^29^,^30^. The mitochondria also produces several antioxidants to balance the production of ROS^31^. Antioxidant down-regulation, in addition with decreased Ox-PC species, suggests decreased ROS formation in the embryo implantation sites of *p53*^*d/d*^ mice. *Trp53* deficiency has been shown to decrease mitochondrial ROS in previous studies^32^,^33^.

The Ox-PC species, here reported as depleted in the *p53*^*d/d*^ tissue, are spontaneously produced by the reaction between ROS and an unsaturated acyl chain, such as linoleic acid (FA 18:2) or arachidonic acid (FA 20:4)^34^. PC species with FA 20:4 in the sn-2 position can be oxidized to yield HETE-PC or be both oxidized and truncated to form HOOA-PC^20^. The Ox-PC species ON-PC is generated and truncated by the oxidation of a PC containing an FA 18:2 in the sn-2 position^20^,^34^. A second oxidation of ON-PC generates Az-PC^20^. Since two oxidation processes are needed to form Az-PC, a high abundance of ROS is essential for the formation of Az-PC. In this study, Az-PC is one of the most significantly depleted Ox-PC species. We propose that the significant decrease of Az-PC in *p53*^*d/d*^ is a result of decreased abundance of ROS and impaired mitochondrial β-oxidation in the *p53*^*d/d*^ implantation sites on day 8 of pregnancy.

Lipid peroxidation is considered to be both beneficial and deleterious in biological systems^35^. Ox-PC species have been found to regulate sterol synthesis, inflammation, angiogenesis, apoptosis, and cell division in endothelial cells^19^. Furthermore, Ox-PC has been shown to induce transcription of anti-oxidant enzymes^36^, interact with the immune system^34^,^36^,^37^,^38^, stimulate Peroxisome Proliferator-Activated Receptor gamma (PPARγ), affect intracellular messaging^34^,^36^, clear cellular debris^37^, and induce apoptosis^34^,^37^,^39^. Lower amounts of COX-2 and prostaglandin E2 have been shown to be linked with increased amounts of lipid peroxides^40^. Therefore both prostaglandins (e.g., PGF_2α_) and COX-2, which have been previously reported to be up-regulated in the uterine tissue of *p53*^*d/d*^ mice^3^, are linked to the lower abundance of Ox-PC in the *p53*^*d/d*^ implantation sites.

Controlled inflammation, i.e. involving prostaglandins, is a major component in pregnancy and is required both for implantation and parturition^41^. During inflammation the oxidative pressure is increased and many oxygen specific epitopes are pro-inflammatory^37^. ROS are known to play roles during embryonic, fetal and placental development^42^,^43^,^44^. Additionally, in clinical studies, low levels of ROS were found in human IVF patients that did not become pregnant^43^ and low levels of antioxidants have resulted in either miscarriage or no pregnancy at all^29^,^42^,^43^. Our results of lower levels of Ox-PC, together with previous results of mitochondrial dysfunction in the *p53*^*d/d*^ implantation sites, support the role of ROS in successful pregnancy. Additionally, our results show that Ox-PC species detected by nano-DESI MSI can be used as molecular markers to follow spatially and temporally resolved progress of mitochondrial β-oxidation throughout pregnancy.

All major lipid groups characterized in this study (FA, MG, DG, and PC) show a relative increase in PUFAs and PUFAs acyl chains in the *p53*^*d/d*^ implantation sites as compared to the *p53*^*f/f*^ implantation sites. This relatively higher abundance of unsaturated lipids in the *p53*^*d/d*^ implantation sites detected in this study is linked with the lower abundance of ROS shown by the depletion of Ox-PC in the *p53*^*d/d*^ implantation sites. This is additionally linked to previous studies showing that cells incubated with PUFAs accumulate DG^45^. The control of membrane PUFA composition is multifactorial. Cellular phospholipids have been shown to incorporate FA 20:5 to a higher degree than FA 18:2, suggesting preferential incorporation of PUFAs into phospholipids^46^. In addition, the expression of specific acyl-CoA synthetase, lysophospholipid acyltransferase and phospholipase A2 isotypes have an impact on membrane PUFA content^47^. An increase in abundance of membrane PUFA content results in increased membrane fluidity and decreased membrane thickness, which may influence cellular events^45^,^48^. Additionally, due to the interdependence of biologically active lipid mediators and their precursor membrane lipids, regional changes in membrane PUFA content can provide novel insight into cell-type-specific lipid singling. For example, in this study we characterized accumulation of PC 36:4 (16:0–20:4) and arachidonic acid (FA 20:4) to the decidua of *p53*^*d/d*^ implantation sites, suggesting a possible role of PC 36:4 in decidua-derived cytosolic phospholipase A_2α_‒COX-2‒PGF_2α_ signaling^18^. Due to the low endogenous concentration of many lipid mediators, cutting edge analytical technologies used to characterize these molecules still require bulk analysis techniques using homogenization which can obscure detailed information on the spatial localization of these molecules to the heterogeneous cell types of the uterus during early pregnancy. As illustrated in this nano-DESI MSI study and our previous MALDI MSI study^18^, characterizing novel spatiotemporal lipid changes during embryo implantation in their pleomorphic roles depend on their proximity to the implanting embryo and with pregnancy progression. MSI has the unparalleled ability to uncover the complexity of lipid homeostasis in heterogeneous uterine tissue during normal and perturbed embryo implantation^11^,^12^,^18^,^49^. Future studies establishing detailed mechanistic connections between specific membrane PUFA containing lipids and their metabolism into biologically active lipid mediators is essential for advancing our understanding of specific lipid pathways during early pregnancy and for devising novel strategies for management and treatment in perturbed systems such as preterm birth.

In summary, *Trp53* deficiency influences lipid signaling and homeostasis in *p53*^*d/d*^ implantation sites contributing to preterm labor. This study demonstrates for the first time that *Trp53* deficiency results in aberrant global changes in the amount and localization of several types of lipids at the implantation site. Additionally, this is the first study to use MSI to evaluate Ox-PC changes within a biological context. The significant lipid changes presented here include an overall increase in PUFAs, an increase of multiple DG species, linked to an increase in lipid mediator signaling and resulting from increased PPAP2A expression, and a decrease in multiple Ox-PC species resulting from decreased ROS formation due to decreased mitochondrial β-oxidation and ATP-production. Characterizing abnormal changes in the abundance and cellular localization of individual lipid species early in the progression to premature birth is an essential step toward understanding the pathophysiology of preterm birth and potentially discovering novel targets for treatments and diagnosis.

## Methods

Herein we employed nano-DESI MSI technology to define alterations in lipid classes and their molecular species, while maintaining the spatial integrity for the heterogeneous cell types of the uterus in implantation sites collected on day 8 of pregnancy from three *p53*^*f/f*^ and three *p53*^*d/d*^ mice. From each of our 6 mice, successive 12-μm thick sections from the center of an implantation site were mounted onto glass slides; each slide contained 16 to 20 sections per implantation site. For each experiment, 3–4 center-sections were analyzed resulting in 10 *p53*^*d/d*^ and 11 *p53*^*f/f*^ positive mode ionization nano-DESI images and 9 *p53*^*d/d*^ and 10 *p53*^*f/f*^ negative mode ionization nano-DESI images. Statistical comparisons of these ion images were performed on mass spectral abundance data extracted from the M-pole and AM-pole using specific ions as markers for the regions^18^. In positive mode the AM-pole region was marked by *m/z* 848.56 and the M-pole was marked by *m/z* 798.54, similarly *m/z* 790.53 marked the AM-pole in negative mode and *m/z* 883.52 marked the M pole region. Our high quality mass spectrometry images were in part due to spiking internal standards into the nano-DESI solvent to generate ion images free of matrix effects showing the actual localization of the endogenous lipid species. In addition, comparisons with littermate mice ensured that data was not confounded by variations in genetic backgrounds. See Supporting Information for detailed methods. All experiments for the present study were conducted in accordance with the guidelines of the National Institutes of Health and were approved by the Cincinnati Children’s Research Foundation Institutional Animal Care and Use Committee.

### Nano-DESI MSI

Embryo implantation sites mounted on glass slides were stored at −80 °C and were equilibrated to room temperature prior to analysis; appropriate sample handling limited lipid degradation^50^. Nano-DESI MSI was performed as described elsewhere^11^. In short, two fused silica capillaries (I.D 50 μm, O.D 150 μm, from Polymicron Technologies) were positioned at an angle to each other in from of the mass spectrometer inlet. Data was acquired in full scan mode (*m/z* 100–2000) with automated gain control using an LTQ-Orbitrap XL (Thermo Scientific) mass spectrometer. Ion images were acquired in both positive and negative mode, from separate implantation site sections, at a mass resolution of either 60 000 or 100 000 (*m/*Δ*m*). The nano-DESI solvent for positive mode consisted of LPC 19:0, PE 15:0/15:0, PG 15:0/15:0, PS 17:0/17:0, PC 23:0/23:0 and DG 14:0/14:0 (all from Avanti Polar Lipids) at the respective concentrations 0.18 μM, 7.2 μM, 4.8 μM, 18 μM, 45 μM, 2.1 μM, in methanol:water (9:1, v-v). The nano-DESI solvent for negative mode consisted of PE 15:0/15:0, PG 15:0/15:0 and PS 17:0/17:0 at the respective concentrations 5.0 μM, 0.15 μM and 1.25 μM, in methanol:water (9:1, v-v). The solvent was continuously delivered at 0.5 μL/min, regardless of polarity, using a syringe pump (Legato 180, KD Scientific). The solvent maintained a liquid bridge between the two capillaries into which molecules from the sample surface were desorbed. A high voltage at 3.0 kV for positive mode and 2.5 kV for negative mode was applied to the primary capillary and the secondary capillary functioned as the electrospray emitter. The sample was mounted on a motorized XYZ translational stage operated by a custom-designed LabVIEW software and moved in z-direction^10^. The stage was continuously moved under the nano-DESI probe at 20 μm/s when data was acquired at 100 000 (*m/*Δ*m*) and at 40 μm/s when acquiring at 60 000 (*m/*Δ*m*). Lines were spaced by 150 μm resulting in an average pixel size of approximately 12 × 150 μm^2^ (x × y) for ion images recorded at mass resolution of 100 000 and 40 × 150 μm^2^ (x × y) for ion images recorded at mass resolution of 60 000. To ensure independence of carry over effects between implantation sites regions, the direction of analysis was alternated, from AM-pole to M-pole or from M-pole to AM-pole, between biological replicates.

Ion images were generated using the in-house developed software MSIQuickview^10^. The presented ion images of Ox-PC species (Fig. 3) are normalized to the standard LPC 19:0 (0.18 μM) to account for matrix effects which could distort the ion distribution^13^. Similarly, the presented ion images of DG species (Fig. 2) are normalized to the standard DG 14:0/14:0/0:0 (2.1 μM). The ion images of abundant PC species (Fig. 1) were normalized to the total ion current. Each ion image has its own intensity scale (0–100%) to increase clarity in presentation. Tables S2–S4 detail the *m/z*, p-values, and abundance data depicted in Figs 1, 2, 3. For Fig. 4, comparison of molecular species within each lipid class was performed by normalizing the signal for each species within the ROI with the signal for the sum of all species within the molecular class. Table S5 details the *m/z*, p-values, abundance data, and the ratio of the *p53*^*d/d*^ over *p53*^*f/f*^ which were calculated to show the differences depicted in Fig. 4.

### Peak assignment

DG species are assigned based on 1) their *m/z* 2) the existence of multiple cation adducts 3) their ionization properties (similarly to the standard they do not produce negative ions) and 4) the lack of MS/MS fragments supported by the need of high collision energy for fragmentation of the standard. Phosphatic acid (PA) 17:0/17:0 (Avanti Polar Lipids) and DG 14:0/14:0/0:0 (Avanti Polar Lipids) was used to determine differences in ionization and fragmentation between PA and DG to further confirm the identity of endogenous DG; due to the close *m/z* range for accurate masses of DG species and PA species. See supporting information. Oxidized phosphatidylcholines are assigned based on 1) their *m/z* 2) MS/MS. See supporting information.

## Additional Information

**How to cite this article**: Lanekoff, I. *et al. Trp53* deficient mice predisposed to preterm birth display region-specific lipid alterations at the embryo implantation site. *Sci. Rep.* **6**, 33023; doi: 10.1038/srep33023 (2016).

## Supplementary Material

Supplementary Information
