# Supplementary material for: Trp53 deficient mice predisposed to preterm birth display region-specific lipid alterations at the embryo implantation site
Source: Sci Rep. 2016 Sep 13;6:33023. doi: 10.1038/srep33023 (PMC5020559; doi:10.1038/srep33023)
Supplement: Supplementary Information [file srep33023-s1.pdf]

Supporting information

Trp53 deficient mice predisposed to preterm birth display region-specific lipid alterations at the embryo implantation site

Ingela Lanekoff[a,b], Jeeyeon Cha[d^], Jennifer E. Kyle[c], Sudhansu K. Dey[d], Julia Laskin[a], Kristin E. Burnum-Johnson[c]*

[a]Physical Sciences Division, Pacific Northwest National Laboratory, Richland, WA, 99352
[b]Department of Chemistry-BMC, Uppsala University, Sweden
[c]Biological Sciences Division, Pacific Northwest National Laboratory, Richland, WA, 99352
[d]Division of Reproductive Sciences, Cincinnati Children's Hospital Medical Center, Cincinnati, OH 45229

[^]Current address: Department of Medicine, Vanderbilt University, Nashville, TN

*To whom correspondence should be addressed: Kristin E. Burnum-Johnson, Pacific Northwest National Laboratory, Richland WA 99352
E-mail: kristin.burnum-johnson@pnnl.gov

Method details

Mice. *Trp53*$^{loxP/loxP}$ *Pgr*$^{+/+}$ (*p53*$^{f/f}$) and *Trp53*$^{loxP/loxP}$ *Pgr* $^{Cre/+}$ (*p53*$^{d/d}$) females were generated as previously described (1). All protocols for the present study were reviewed and approved by the Cincinnati Children's Research Foundation Institutional Animal Care and Use Committee in accordance with National Institutes of Health guidelines. For experiments, littermate *p53*$^{f/f}$ and *p53*$^{d/d}$ mice were used. Mice were provided with autoclaved rodent LabDiet 5010 (Purina) and UV light–sterilized RO/DI constant circulation water ad libitum and were housed under a constant 12-hour light/12-hour dark cycle.

Tissue collection. Adult *p53*$^{f/f}$ and *p53*$^{d/d}$ female mice were mated with fertile CD1 males to induce pregnancy (day 1 = vaginal plug). One embryo implantation site on day 8 of pregnancy was isolated from each of 3 *p53*$^{f/f}$ and 3 *p53*$^{d/d}$ uteri and snap frozen. Successive 12-μm thick sections from the central part of the implantation site were mounted onto glass slides precoated with poly-L-lysine; each slide contained 16 to 20 sections per implantation site. Slides were stored at −80 °C and were equilibrated to room temperature prior to analysis; appropriate sample handling limited lipid degradation (2). 3-4 center-sections were analyzed.

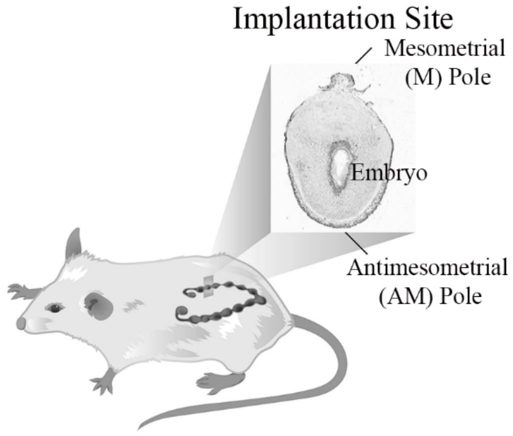

Experimental Design: The nano-DESI mass spectrometry imaging (MSI) lipid profiles of 3 *p53*$^{f/f}$ and 3 *p53*$^{d/d}$ mice were compared by analyzing 3-4 center-sections from one implantation site per dam on day 8 of pregnancy.

Nano-DESI MSI. Nano-DESI MSI was performed using fused silica capillaries (50 id and 150 od) assembled in a capillary holder (described elsewhere (3)). Data was acquired in full scan mode (*m/z* 100-2000) with automated gain control using an LTQ-Orbitrap XL (Thermo Scientific) mass spectrometer. Ion images were acquired in both positive and negative mode, from separate implantation site sections, at a mass resolution of either 60 000 or 100 000 ($m/\Delta m$). The nano-DESI solvent for positive mode consisted of LPC 19:0, PE 15:0/15:0, PG 15:0/15:0, PS 17:0/17:0, PC 23:0/23:0 and DG 14:0/14:0 (all from Avanti Polar Lipids) at the respective concentrations 0.18 μM, 7.2 μM, 4.8 μM, 18 μM, 45 μM, 2.1 μM, in methanol:water (9:1, v:v). The nano-DESI solvent for negative mode consisted of PE 15:0/15:0, PG 15:0/15:0 and PS 17:0/17:0 at the respective concentrations 5.0 μM, 0.15 μM and 1.25 μM, in methanol:water (9:1, v:v). The solvent was continuously delivered at 0.5 μL/min, regardless of polarity, using a syringe pump (Legato 180, KD Scientific). A high voltage at 3.0 kV for positive mode and 2.5

kV for negative mode was applied to the primary capillary. The sample was mounted on a motorized XYZ translational stage operated by a custom-designed LabVIEW software and moved in z-direction according to the plane in which the sample resided (described elsewhere)(4). The stage was continuously moved under the nano-DESI probe at 20 µm/s when data was acquired at 100 000 ($m/\Delta m$) and at 40 µm/s when acquiring at 60 000 ($m/\Delta m$). Lines were spaced by 150 µm resulting in an average pixel size of approximately 12×150 μm^2 (x×y) for ion images recorded at mass resolution of 100 000 and 40×150 μm^2 (x×y) for ion images recorded at mass resolution of 60 000. To ensure independence of carry over effects between implantation sites regions, the direction of analysis was alternated, from AM-pole to M-pole or from M-pole to AM-pole, between biological replicates.

Data processing: Ion images were generated using the in-house developed software MSIQuickview(4). The presented ion images of Ox-PC species, depicted in **Figure 3** in the manuscript, are normalized to the standard LPC 19:0 (0.18 µM) to account for matrix effects which could distort the ion distribution(5). Similarly, the presented ion images of DG species, depicted in **Figure 2** in the manuscript, are normalized to the standard DG 14:0/14:0/0:0 (2.1 µM). The ion images of abundant PC species, depicted in **Figure 1** in the manuscript, were normalized to the total ion current. Each ion image has its own intensity scale (0-100%) to increase clarity in presentation. Note that the higher ion signals at the embryonic site are interpreted to be an artifact of analysis originating from the more compact material at this site. **Tables S2-S4** detail the *m/z*, p-values, and abundance data depicted in **Figures 1, 2 & 3**. For **Figure 4**, Comparison of molecular species within each lipid class was performed by normalizing the signal for each species within the region of interest with the signal for the sum of all species within the molecular class. **Table S5** details the *m/z*, p-values, abundance data, and the ratio of the $p53^{d/d}$ over $p53^{f/f}$ which were calculated to show the differences depicted in **Figure 4** in the manuscript.

Regions of interest were generated using MSIQuickview(4) using specific ions as markers for the regions. In positive mode the AM-pole region was marked by *m/z* 848.56 and the M-pole was marked by *m/z* 798.54, similarly *m/z* 790.53 marked the AM-pole in negative mode *m/z* 883.52 marked the M pole region.

Peak assignment: DG species are assigned based on 1) their *m/z* 2) the existence of multiple cation adducts 3) their ionization properties (similarly to the standard they do not produce negative ions) and 4) the lack of MS/MS fragments supported by the need of high collision energy for fragmentation of the standard. Phosphatic acid (PA) 17:0/17:0 (Avanti Polar Lipids) and DG 14:0/14:0/0:0 (Avanti Polar Lipids) was used to determine differences in ionization and fragmentation between PA and DG to further confirm the identity of endogenous DG; due to the close *m/z* range for accurate masses of DG species and PA species. Oxidized phosphatidylcholines are assigned based on 1) their *m/z* 2) MSMS. (See supporting information below).

RNA isolation and quantitative PCR. RNA was prepared from homogenized implantation sites using TRIzol reagent (Invitrogen). RNA extraction was performed as described previously(1, 6). Quantitative PCR (qPCR) was performed using StepOnePlus Real-Time PCR System (Applied Biosciences). PCR was performed using the following primers: 5'-AGGAAGAGGATCCACACACG - 3'and 5'- TGGGCATCCAGGAGGTATAG - 3' for *Ppap2a*; 5' – TTTGGCCTTTTGCTCTCCTA - 3' and 5'- ATGCTGTGGATCTGGAAAGG - 3' for *Cds2*; and 5'-TCCATGACAACTTTGGCATTG - 3′ and 5′ - CAGTCTTCTGGGTGGCAGTGA - 3' for *Gapdh*. *Gapdh* served as a housekeeping gene for mouse tissues.

Statistics of Biological Experiments. Statistical analyses were performed using 2-tailed heteroscedastic Student's *t*-test. *P*-values below 0.05 were considered statistically significant. All p-values for reported results can be found in **Tables S2-S5** below.

Peak assignment of DIACYLGLYCEROL species

Despite high mass resolution mass spectrometry some peaks identified as diacylglycerol by accurate mass could potentially be assigned to plasmalogen species of phosphatic acid, as shown in **Table S1**. **Table S1** includes protonated, sodiated and potassiated species possible within 10 ppm of the experimentally obtained *m/z* values. In nano-DESI MSI of tissue from mouse embryo implantation sites, the majority of chemical species are found as potassium adducts. However, no potassium adducts are found for any of the suggested PA species, but there are sodium adducts found to support the assignment of potassiated DG species (marked by stars in **Table S1**).

The standards DG 14:0/14:0/0:0 and PA 17:0/17:0 were used at high concentrations (100 µM and 133 µM, respectively) to compare ionization and fragmentation patterns of the two lipid classes, PA and DG. It was found that while PA ionizes in both positive and negative mode, as shown in **Figure S1**, DG only ionizes in positive mode as shown in **Figure S2**. Not even at extremely high concentrations (500 µM) did DG produce ions in negative mode. This difference between the two species validates the assignment of DG since no peaks corresponding to the possible endogenous PA species were found in negative mode spectra obtained during MSI. MSMS of the standards further showed that DG requires a higher collisional energy to produce fragment ions. MSMS of the peaks of interest was performed directly from the tissue but no fragments of was found. The difficulty to produce fragment ions further supports the assignment of DG.

Table S1. Diacylglycerol identification * Mark DG species supported by a sodium adduct, no potassium adduct is detected for any PA species

m/z	**[M+H]+**		**[M+Na]+**		**[M+K]+**
**629.4589**			PA(O-30:0)		DG 34:3
**631.4747**		PA(P-32:1)			DG 34:2*
**633.4905**	PA(O-32:1)	PA(P-32:0)			DG 34:1*
**655.4749**	PA(O-34:4)	PA(P-34:3)	PA(O-32:1)	PA(P-32:0)	DG 36:4*
**657.4908**	PA(O-34:3)	PA(P-34:2)	PA(O-32:0)		DG 36:3*
**659.5064**	PA(O-34:2)	PA(P-34:1)			DG 36:2*
**661.522**	PA(O-34:1)	PA(P-34:0)			DG 36:1
**681.4905**	PA(O-36:5)	PA(P-36:4)	PA(O-34:2)	PA(P-34:1)	DG 38:5*
**683.5064**	PA(O-36:4)	PA(P-36:3)	PA(O-34:1)	PA(P-34:0)	DG 38:4*
**685.5227**	PA(O-36:3)	PA(P-36:2)	PA(O-34:0)		DG 38:3
**703.4751**			PA(O-36:5)	PA(P-36:4)	DG 40:8
**705.4916**		PA(P-38:6)	PA(O-36:4)	PA(P-36:3)	DG 40:7
**709.5219**	PA(O-38:5)	PA(P-38:4)	PA(O-36:2)	PA(P-36:1)	DG 40:5

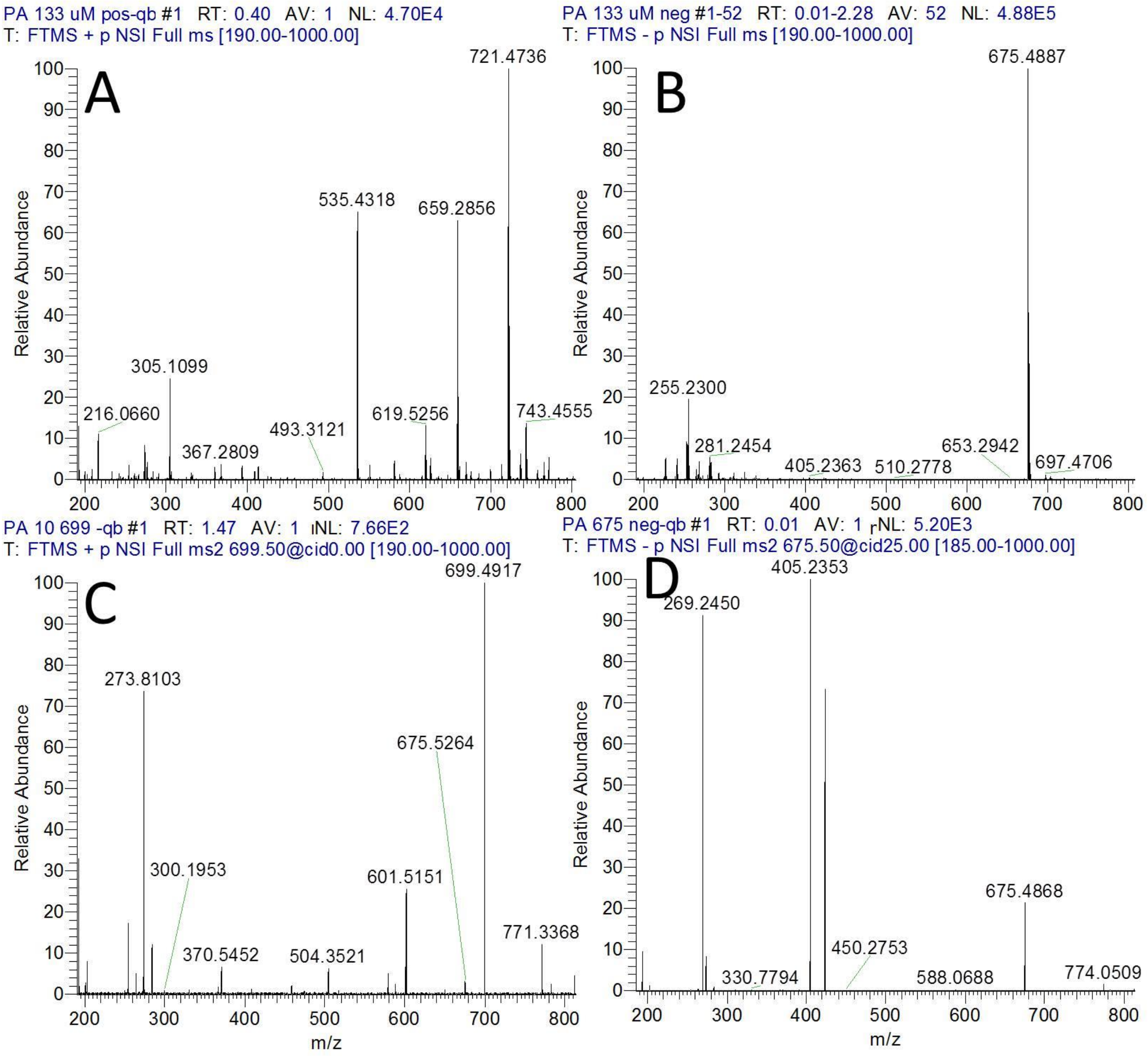

Figure S1. Spectra of standard 133 µM PA 17:0/17:0. A) Positive mode showing $[M+H]^+$ at *m/z* 677.5121, $[M+Na]^+$ at *m/z* 699.494, $[M+K]^+$ at *m/z* 715.4679, $[M+2Na]^+$ at *m/z* 721.4739, $[M+Na+K]^+$ at *m/z* 737.4478. B) Negative mode showing $[M-H]^-$ at *m/z* 675.4965 C) MSMS of $[PA\ 17{:}0/17{:}0+Na]^+$ at *m/z* 699.4922 and a collision energy of 25 showing the fragment ion of 601.5151 corresponding to a neutral loss of the PA head group PO_4H_3. D) MSMS of $[PA\ 17{:}0/17{:}0-H]^-$ at *m/z* 675.4868 showing the fragment ion of *m/z* 269.2450 corresponding to $[FA\ 17{:}0-H]^-$.

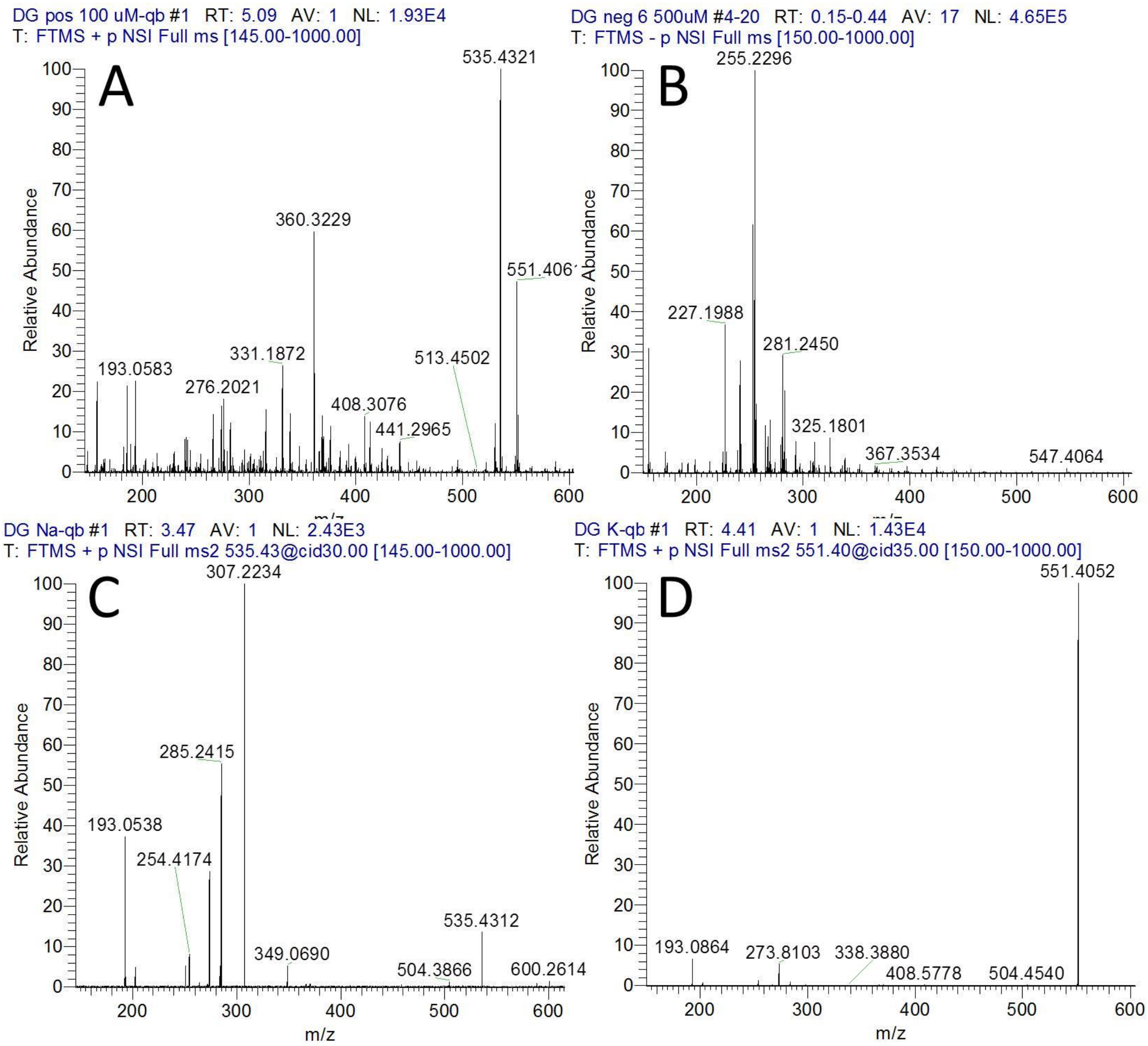

Figure S2. Spectra of standard DG 14:0/14:0/0:0. A) Positive mode showing $[M+H]^+$ at *m/z* 513.4502, $[M+Na]^+$ at *m/z* 535.432, $[M+K]^+$ at *m/z* 551.4061 of 100 µM DG 14:0/14:0/0:0. B) Negative mode without any peaks from 500 µM DG 14:0/14:0/0:0 C) MSMS of $[M+Na]^+$ at *m/z* 535.432 at 100 µM and a collision energy of 30 showing the fragment ion of 251.1975 corresponding to $[FA\ 14:0 + Na]^+$. D) MSMS of $[M+K]^+$ at *m/z* 551.4061 at 100 µM and a collision energy of 35 showing the low amount of fragmentation obtained, fragment ion of *m/z* 267.1624 corresponding to $[FA\ 14:0 + K]^+$.

Peak assignment of OXIDIZED PHOSPHATIDYLCHOLINE species

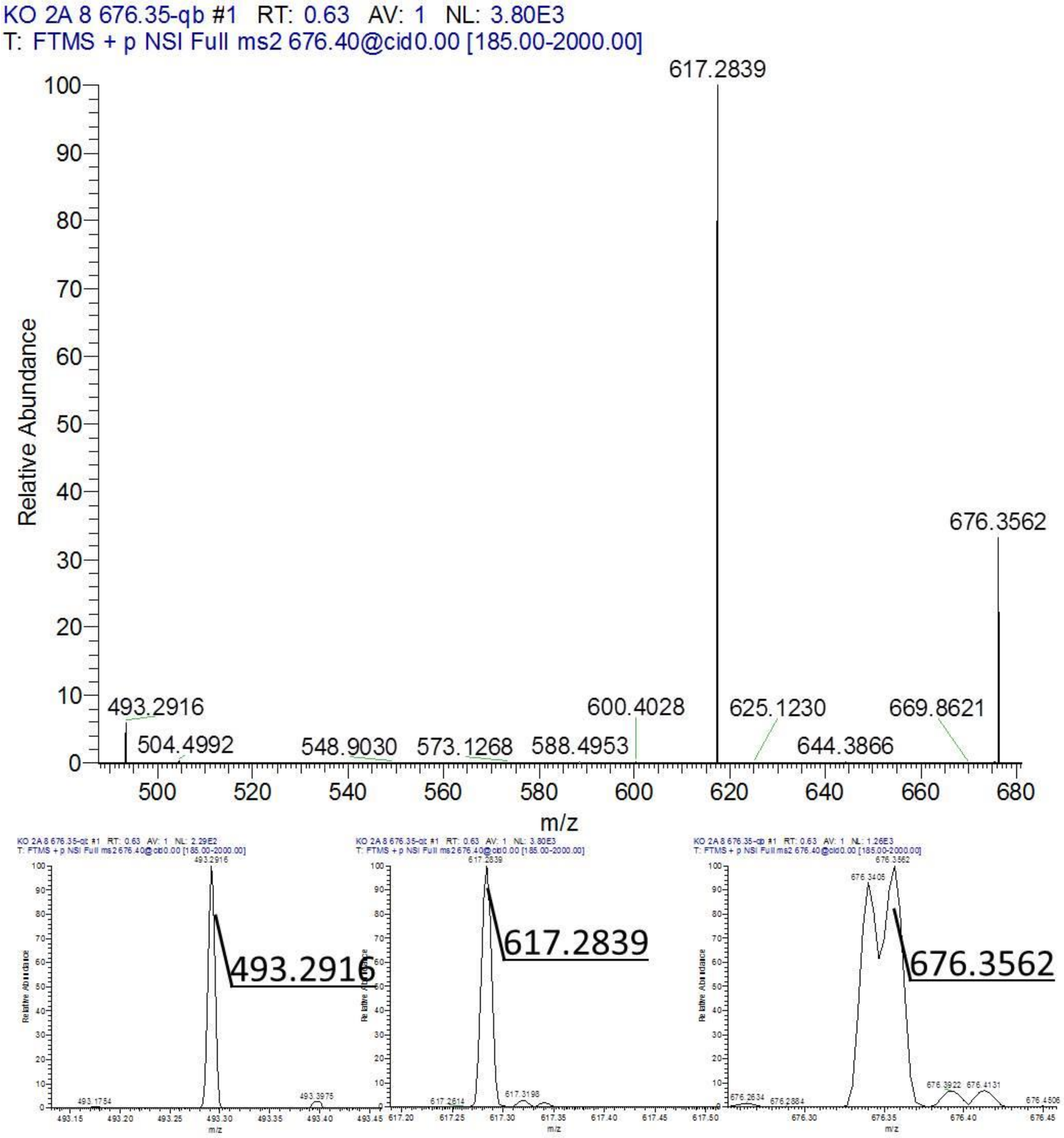

Figure S3. MSMS of HAz 14:1 at *m/z* 676.3594 showing the neutral loss of 59.0723 and 183.0646

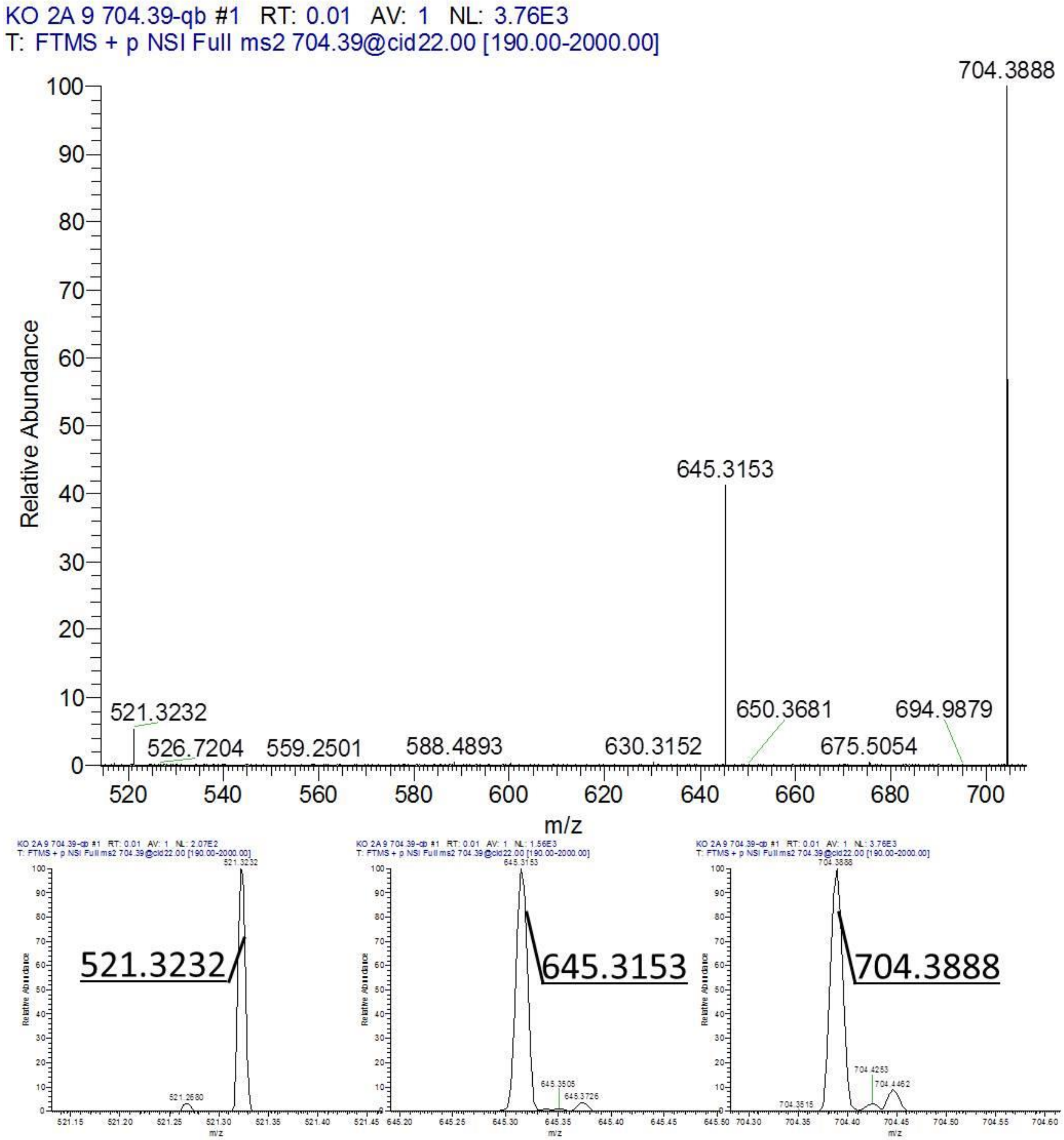

Figure S4. MSMS of HAz 16:0 at *m/z* 704.3907 showing the neutral loss of 59.0735 and 183.0656

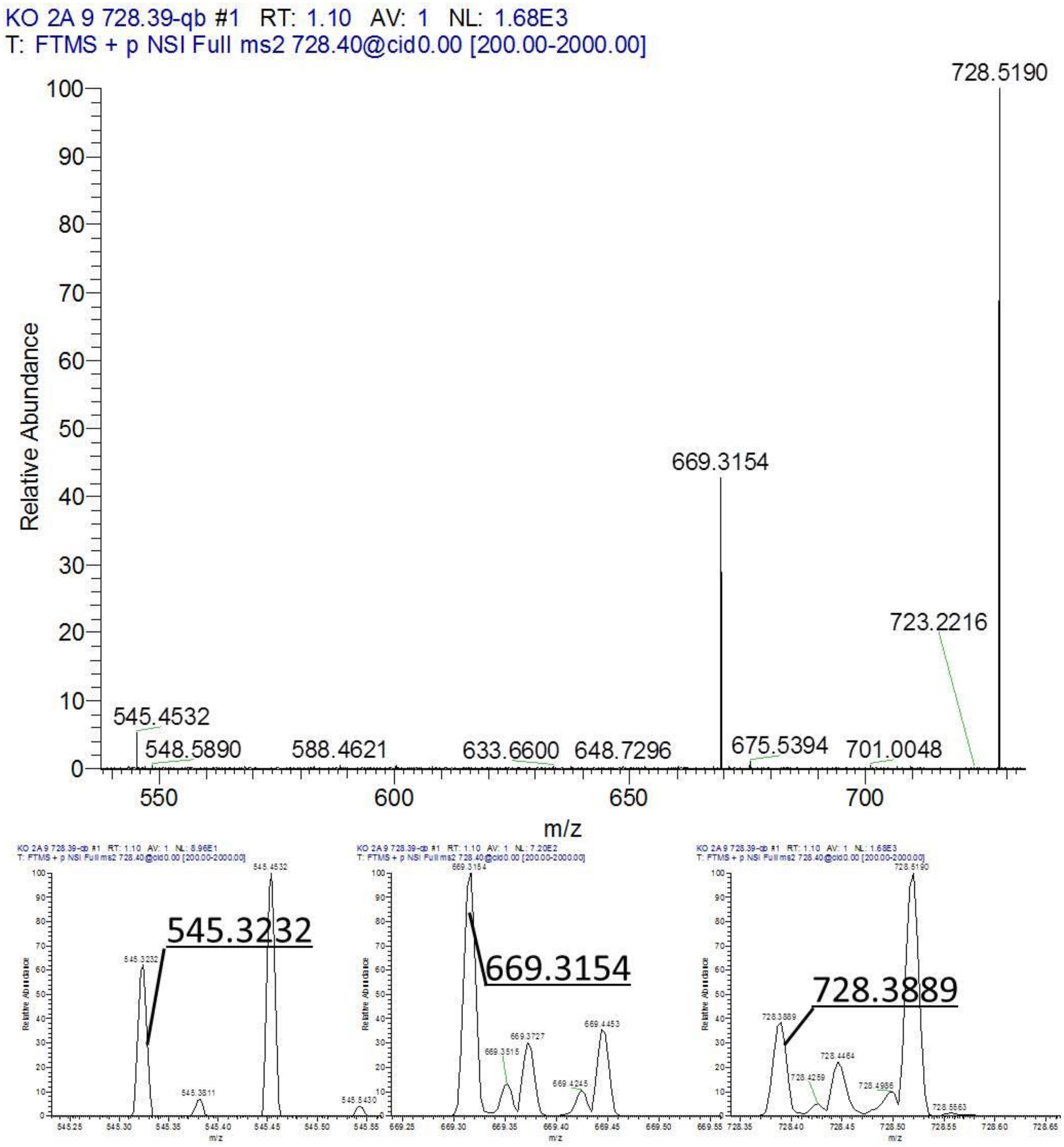

Figure S5. MSMS of HAz 18:2 at *m/z* 728.3907 showing the neutral loss of 59.0734 and 183.0657

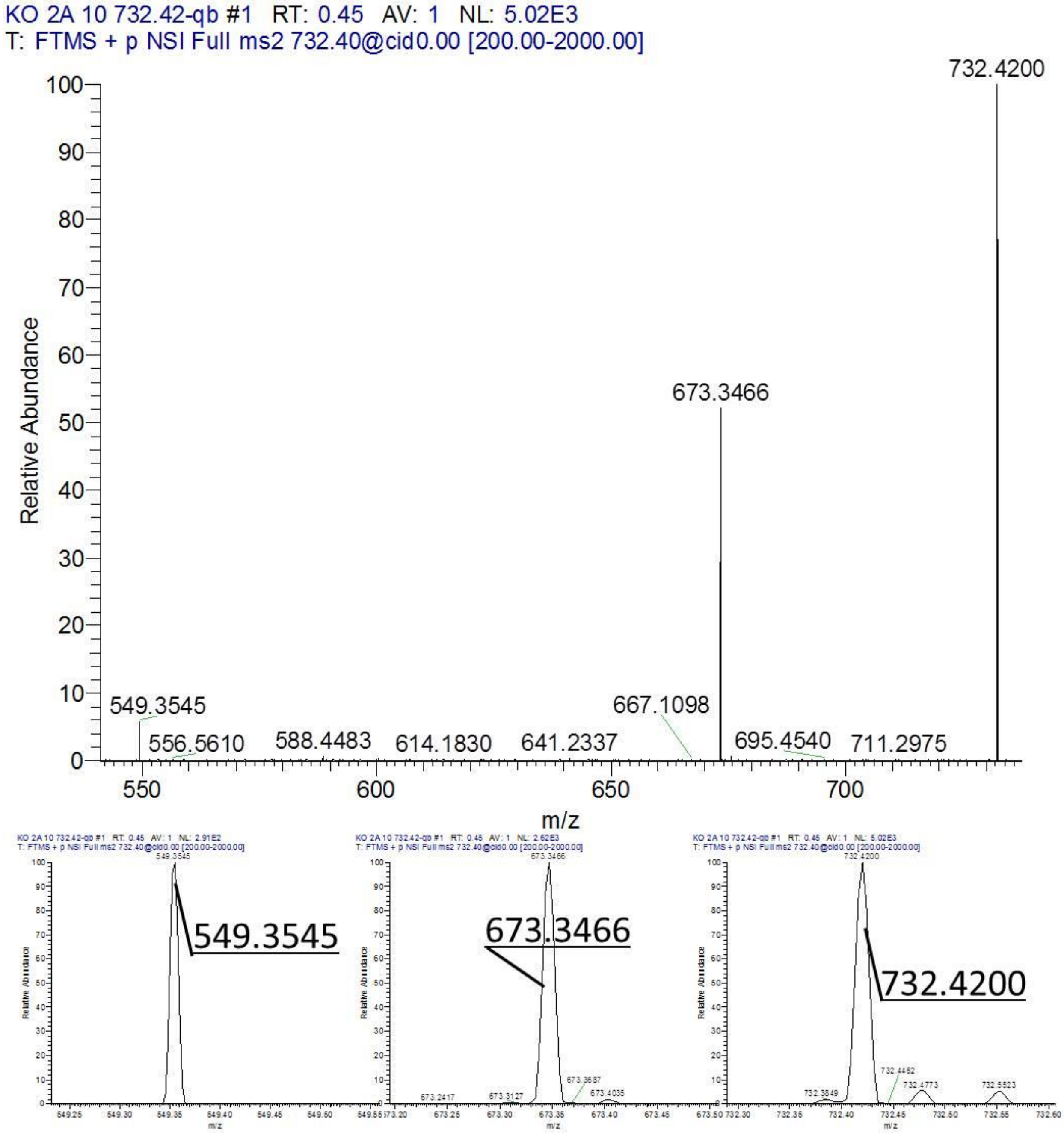

Figure S6. MSMS of HAz 18:0 at *m/z* 732.422 showing the neutral loss of 59.0733 and 183.0655

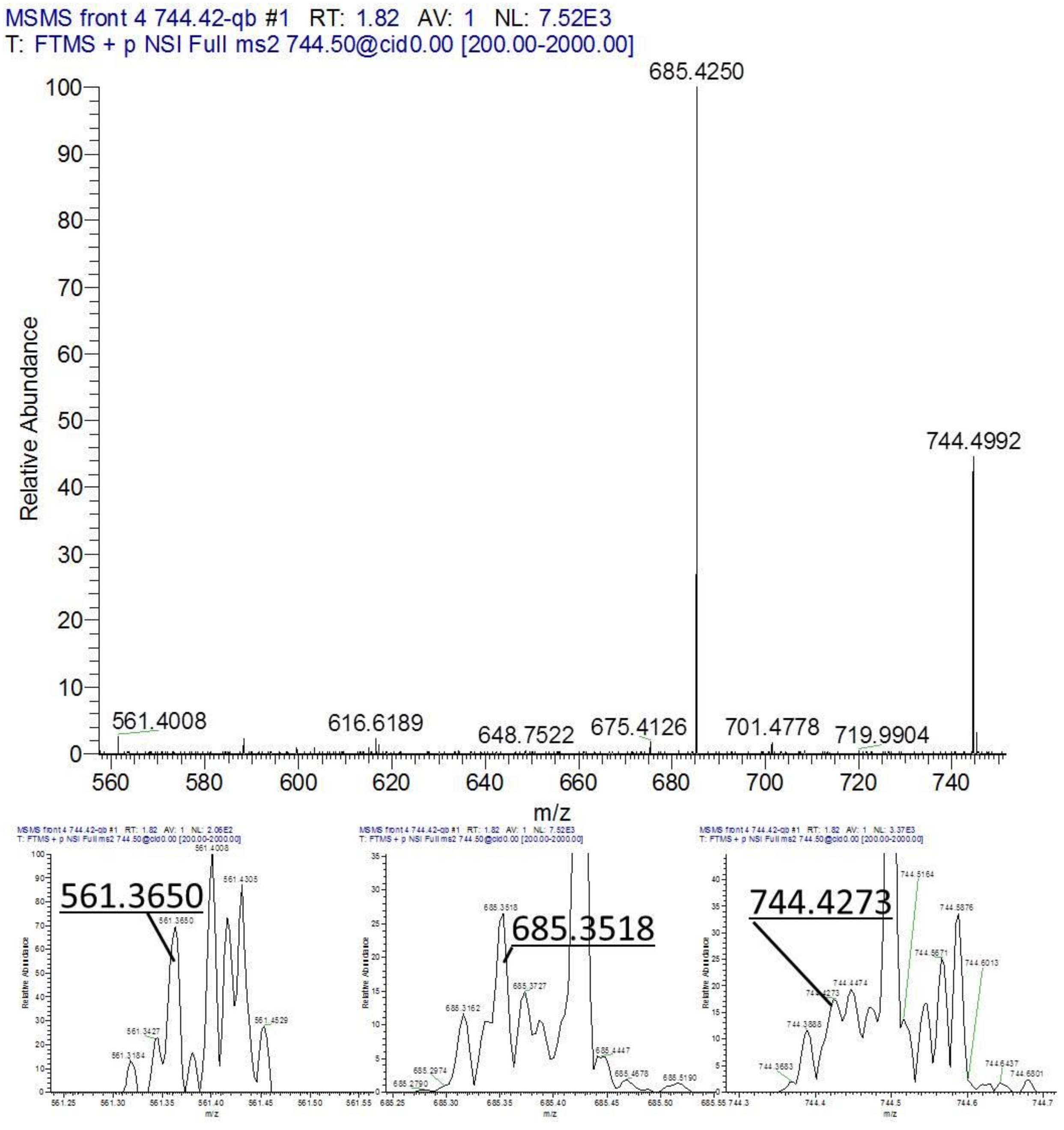

Figure S7. MSMS of HOOA 20:0 at *m/z* 744.4301 showing the neutral loss of 59.0755 and 183.0624

HOOA 22:0 - *m/z* 772.4635

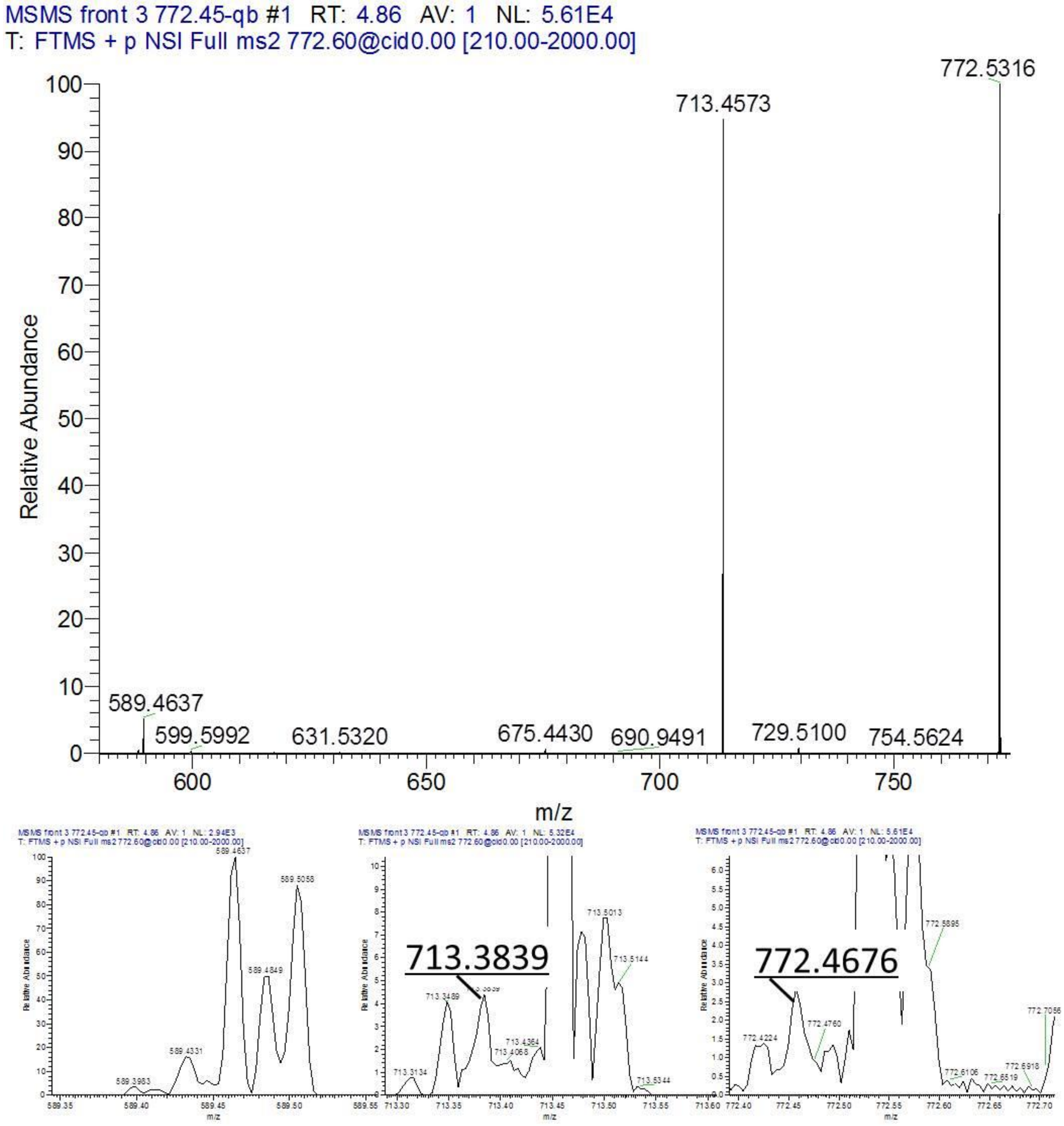

Figure S8. MSMS of HOOA 22:0 at *m/z* 772.4635 showing the neutral loss of 59.0737

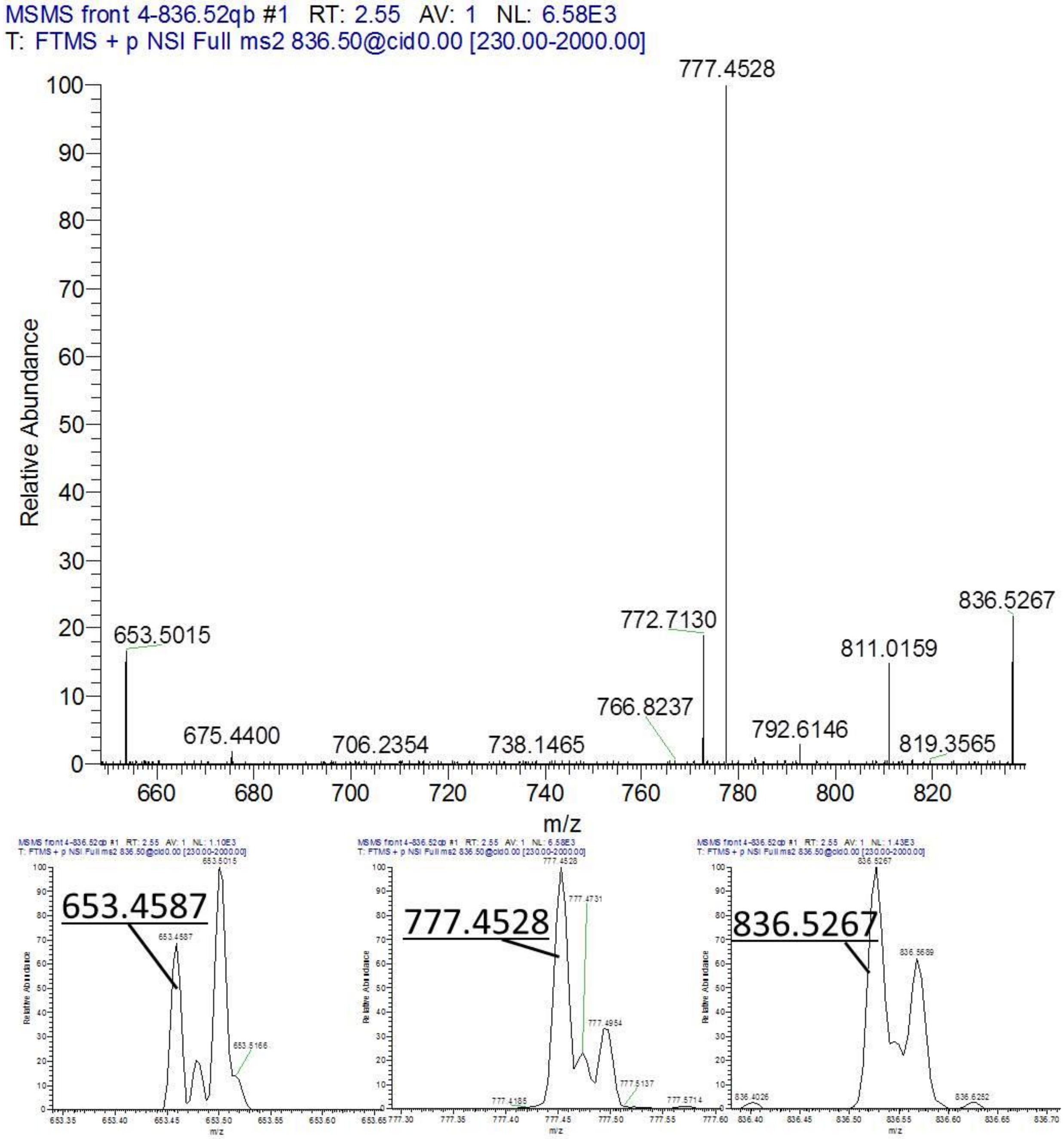

Figure S9. MSMS of HETE 16:0 at *m/z* 836.5267 showing the neutral loss of 59.0739 and 183.0680

HETE 18:0 *m/z* 864.5616

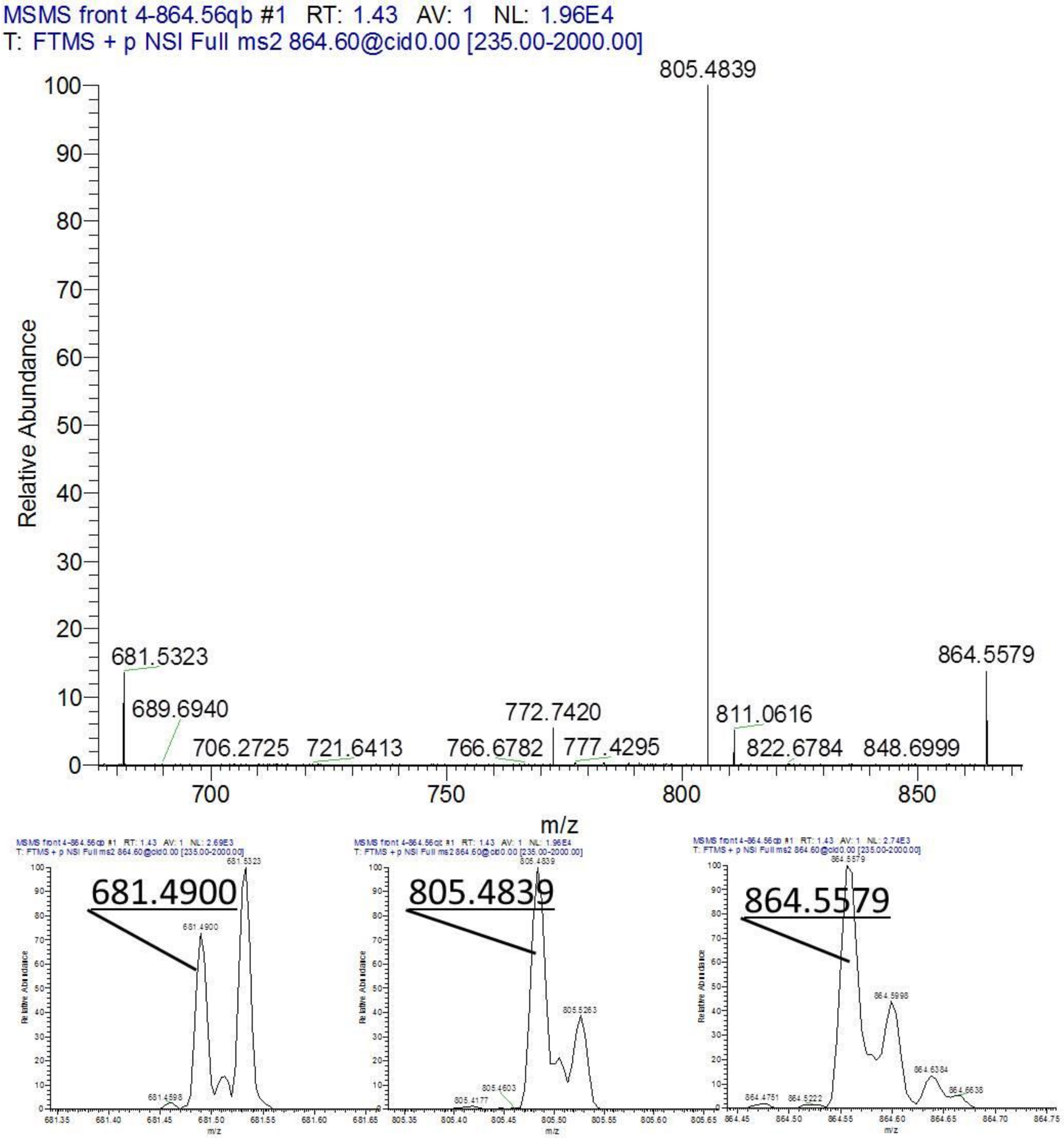

Figure S10. MSMS of HETE 18:0 at *m/z* 864.5616 showing the neutral loss of 59.074 and 183.0679

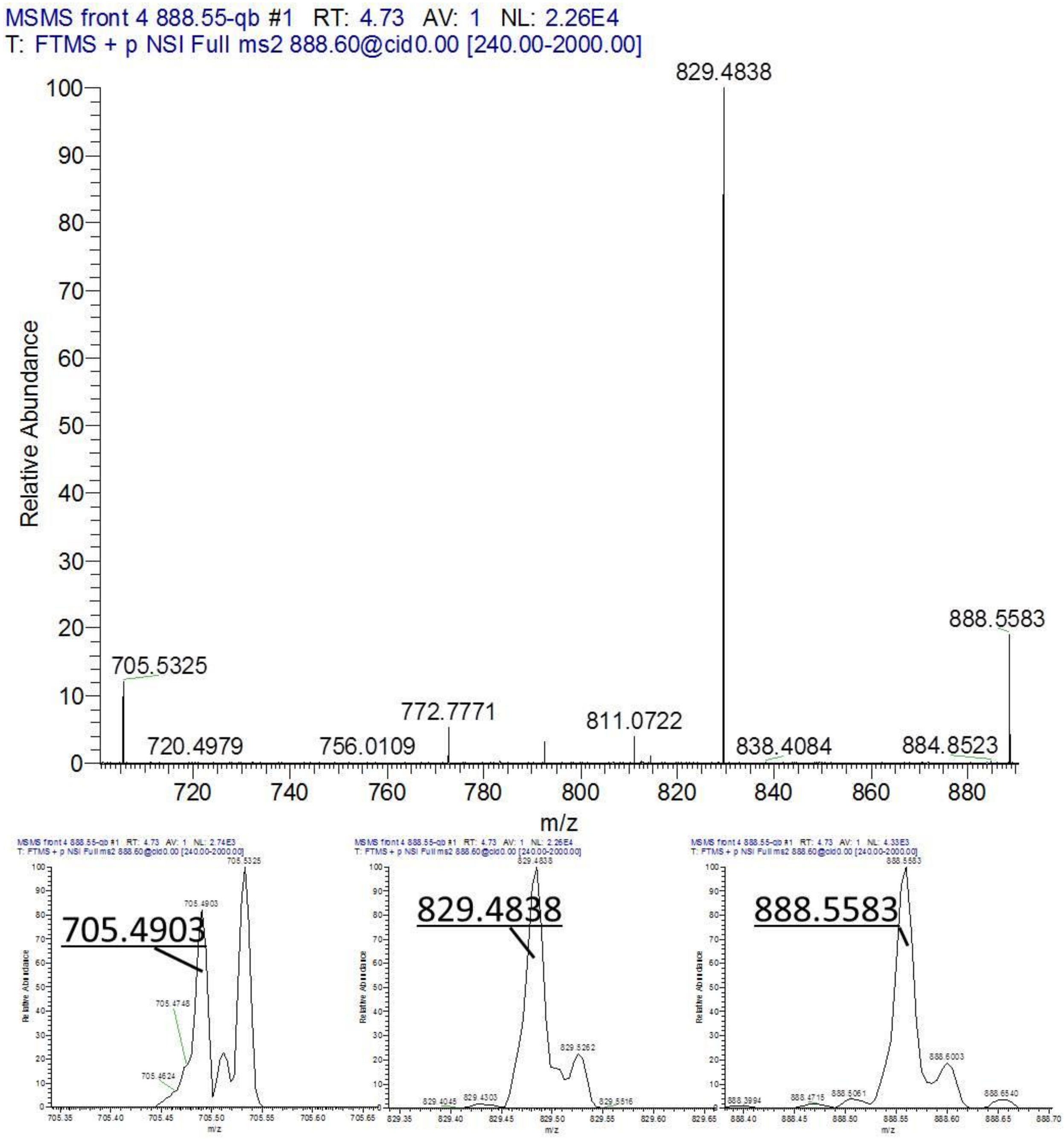

Figure S11. MSMS of HETE 20:2 at *m/z* 888.5608 showing the neutral loss of 59.0745 and 183.0680

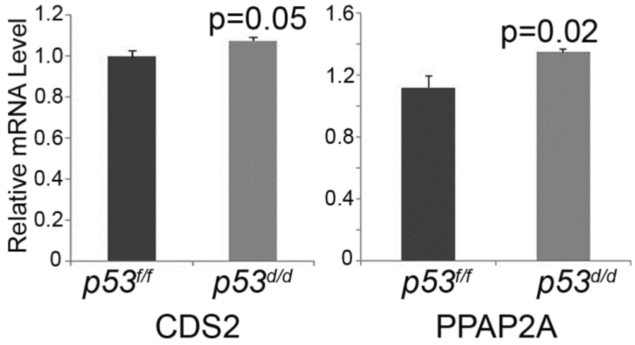

Figure S12. Polymerase chain reaction (qPCR) quantification of phosphatidic acid phosphatase 2a (PPAP2A) and CDP-diacylglycerol synthase (CDS2). qPCR detected a significant increase in enzymes CDS2 and PPAP2A in *p53*$^{d/d}$ vs. *p53*$^{f/f}$ uterine tissue. n=3 females per genotype; mean ± SEM. Only PPAP2A's confident (p-value = 0.02) contribution to DG synthesis was discussed in the manuscript.

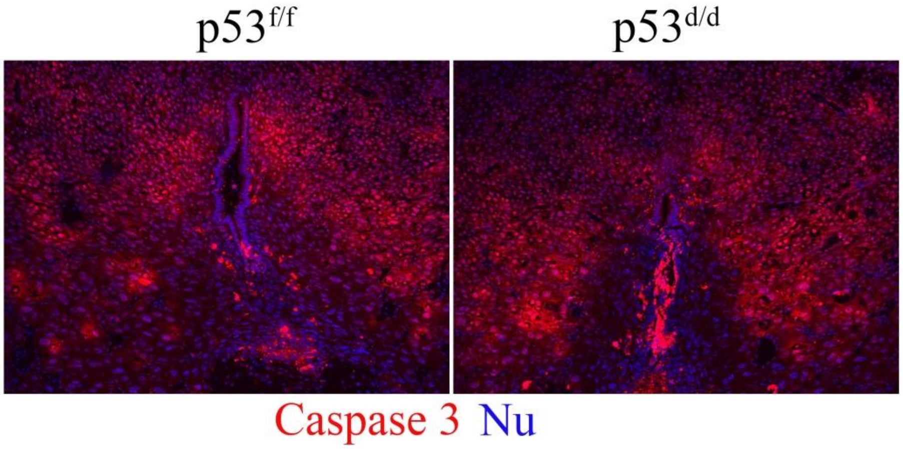

Figure S13. Caspase 3 fluorescence assay of *p53*$^{f/f}$ and *p53*$^{d/d}$ implantation sites showed no significant changes in apoptosis.

Table S2: Data depicted in Figure 1 of the manuscript. Significance as p-values using Student's ttest (heteroscedastic two-tailed distribution), average percent of signal intensities normalized to the total PC pool and standard deviations for PC species. *p-value <0.05; **p-value <0.01

**AM-pole**	*m/z*	P-value	Average *p53d/d*	Average *p53f/f*	Stdev *p53d/d*	Stdev *p53f/f*
PC 30:0	744.5006	0.913	0.6%	0.6%	0.2%	0.2%
PC 32:1	770.5166	0.972	2.0%	2.0%	0.3%	0.2%
PC 32:0	772.5314	0.120	7.3%	7.8%	0.6%	0.8%
PC 34:4	792.5022	0.063	0.0%	0.0%	0.0%	0.0%
PC 34:3	794.5172	0.355	0.6%	0.5%	0.1%	0.1%
PC 34:2	796.5312	0.118	14.1%	13.6%	0.8%	0.5%
PC 34:1	798.5467	0.289	7.0%	7.4%	1.0%	0.5%
PC 36:5	818.516	0.007**	1.0%	0.8%	0.2%	0.1%
PC 36:4	820.5332	0.036*	10.9%	10.4%	0.6%	0.4%
PC 36:3	822.5475	0.652	2.8%	2.9%	0.5%	0.3%
PC 36:2	824.5638	0.688	21.5%	21.7%	1.2%	0.9%
PC 36:1	826.5799	0.768	2.2%	2.2%	0.3%	0.2%
PC 38:6	844.5309	0.448	3.8%	3.7%	0.4%	0.2%
PC 38:4	848.5647	0.299	17.4%	16.9%	1.4%	1.1%
PC 38:3	850.5792	0.419	1.1%	1.2%	0.4%	0.3%
PC 38:2	852.5958	0.063	0.2%	0.1%	0.0%	0.0%
PC 38:1	854.6126	0.546	0.0%	0.0%	0.0%	0.0%
PC 40:8	868.5305	0.329	0.3%	0.2%	0.1%	0.1%
PC 40:7	870.5479	0.371	0.4%	0.5%	0.1%	0.1%
PC 40:6	872.5618	0.051	6.6%	7.4%	1.0%	0.7%
PC 40:4	876.5968	0.539	0.1%	0.1%	0.0%	0.0%
PC 40:3	878.6127	0.675	0.0%	0.0%	0.0%	0.0%
PC 42:7	898.5808	0.086	0.0%	0.0%	0.0%	0.0%
PC 42:6	900.5972	0.041*	0.0%	0.0%	0.0%	0.0%
PC 42:5	902.6144	0.201	0.0%	0.0%	0.0%	0.0%

**M-pole**	*m/z*	P-value	Average *p53d/d*	Average *p53f/f*	Stdev *p53d/d*	Stdev *p53f/f*
PC 30:0	744.5006	0.118	1.0%	1.1%	0.1%	0.2%
PC 32:1	770.5166	0.336	4.8%	5.0%	0.6%	0.5%
PC 32:0	772.5314	0.547	6.0%	5.7%	1.2%	1.0%
PC 34:4	792.5022	0.824	0.1%	0.1%	0.0%	0.0%
PC 34:3	794.5172	0.893	0.8%	0.8%	0.2%	0.1%
PC 34:2	796.5312	0.397	12.8%	13.2%	0.8%	1.1%
PC 34:1	798.5467	0.139	21.4%	22.6%	1.6%	1.7%

PC 36:5	818.516	0.949	0.8%	0.7%	0.2%	0.1%
PC 36:4	820.5332	0.695	7.9%	7.8%	1.3%	0.5%
PC 36:3	822.5475	0.609	6.0%	6.1%	0.6%	0.4%
PC 36:2	824.5638	0.585	15.6%	16.0%	1.3%	1.6%
PC 36:1	826.5799	0.911	5.6%	5.7%	0.9%	0.7%
PC 38:6	844.5309	0.200	2.9%	2.6%	0.6%	0.4%
PC 38:4	848.5647	0.060	8.8%	8.0%	1.1%	0.5%
PC 38:3	850.5792	0.250	1.3%	1.2%	0.1%	0.1%
PC 38:2	852.5958	0.133	0.4%	0.3%	0.0%	0.0%
PC 38:1	854.6126	0.348	0.0%	0.0%	0.0%	0.0%
PC 40:8	868.5305	0.043*	0.2%	0.1%	0.1%	0.0%
PC 40:7	870.5479	0.080	0.6%	0.5%	0.1%	0.1%
PC 40:6	872.5618	0.053	2.5%	2.1%	0.5%	0.4%
PC 40:4	876.5968	0.032*	0.2%	0.2%	0.0%	0.0%
PC 40:3	878.5621	0.350	0.0%	0.0%	0.0%	0.0%
PC 42:7	898.5808	0.019*	0.1%	0.1%	0.0%	0.0%
PC 42:6	900.5972	0.000**	0.1%	0.0%	0.0%	0.0%
PC 42:5	902.6144	0.073	0.0%	0.0%	0.0%	0.0%

Table S3: Data depicted in Figure 2 of the manuscript. Significance as p-values using Student's ttest (heteroscedastic two-tailed distribution), average signal intensities, standard deviations for DG species (none normalized potassium adducts). *p-value <0.05; **p-value <0.01

**AM-pole**	*m/z*	P-value	Average *p53d/d*	Average *p53f/f*	Stdev *p53d/d*	Stdev *p53f/f*
DG 34:3	629.461	0.0054**	2905	987	1656	731
DG 34:2	631.4764	0.0073**	54816	22136	29477	13887
DG 36:4	655.4767	0.0055**	48529	14868	29335	9334
DG 36:3	657.4923	0.0041**	45406	19388	21227	11624
DG 36:2	659.5079	0.0012**	52058	23901	18365	14372
DG 38:5	681.4925	0.0042**	14571	4556	8258	4005
DG 38:4	683.5084	0.0076**	28159	12847	13134	9382
DG 40:8	703.4782	0.0020**	10980	1022	7359	942
DG 40:7	705.4967	0.0016**	9814	1162	6175	1172
DG 40:5	709.5328	0.0115*	3714	1587	2037	1141

**M-pole**	*m/z*	P-value	Average *p53d/d*	Average *p53f/f*	Stdev *p53d/d*	Stdev *p53f/f*
DG 34:2	631.476	0.0109*	19274	7109	9709	10024
DG 34:1	633.492	0.0003**	27403	10302	8375	9212
DG 36:4	655.477	0.0404*	9518	3578	6490	5775

DG 36:3	657.492	0.0028**	18032	5758	8051	8337
DG 36:2	659.508	0.0003**	30755	11298	10318	9443
DG 36:1	661.524	0.0146*	6819	2740	3719	3124
DG 38:4	683.508	0.0058**	15579	6410	7773	4741
DG 40:5	709.533	0.0216*	1460	449	893	957

Table S4: Data depicted in Figure 3 of the manuscript. Significance as p-values using Student's ttest (heteroscedastic two-tailed distribution), average signal intensities, standard deviations for Ox-PC species (none normalized potassium adducts). *p-value <0.05; **p-value <0.01

**AM-pole**	*m/z*	P-value	Average *p53d/d*	Average *p53f/f*	Stdev *p53d/d*	Stdev *p53f/f*
18:0 HOOA	716.3978	0.0168*	5704	17354	5709	12916
20:1 HOOA	742.4146	0.0315*	1578	5040	1676	4430
20:0 HOOA	744.4301	0.0076**	3187	15488	3623	12157
22:0 HOOA	772.4635	0.0115*	5210	27388	7397	23484
14:1 Az	676.3658	0.0083**	17797	59532	22444	39502
16:1 Az	702.3842	0.0085**	985	4696	1370	3704
16:0 Az	704.3948	0.0068**	22611	87698	26523	61955
18:2 Az	728.3984	0.0366*	2333	7152	2461	6368
18:0 Az	732.43	0.0087**	34058	121485	40240	85824
16:0 HETE	836.5296	0.0163*	11195	26812	7107	17359
18:1 HETE	862.547	0.0202*	4952	12310	4163	8323
18:0 HETE	864.5616	0.0270*	16135	36805	13139	24466
20:2 HETE	888.5608	0.0095**	5399	14614	5507	8720
16:0 NO	688.4021	0.0323*	24995	43843	14068	22393

**M-pole**	*m/z*	P-value	Average *p53d/d*	Average *p53f/f*	Stdev *p53d/d*	Stdev *p53f/f*
18:0 HOOA	716.3978	0.0097**	1207	5350	1475	4237
20:0 HOOA	744.4301	0.0067**	1082	6347	1835	5052
22:0 HOOA	772.4635	0.0045**	732	5744	1250	4548
16:0 HETE	836.5296	0.0274*	5154	12387	5406	8190
18:1 HETE	862.547	0.0440*	2053	4854	2650	3285
14:1 Az	676.3658	0.0045**	6424	31699	7340	22864
16:1 Az	702.3842	0.0423*	360	1258	553	1203
16:0 Az	704.3948	0.0060**	18803	92357	19604	69987
18:2 Az	728.3984	0.0189*	546	3210	717	3130
18:0 Az	732.43	0.0067**	16158	71629	18152	53421

Table S5: Data depicted in Figure 4 of the manuscript. Significance in p-values calculated using Student's ttest (heteroscedastic two-tailed distribution), average values after normalization to the total pool of the respective species, standard deviations for signals of lipid species with in the lipid pool of each molecular class and signal ratios of $p53^{d/d}$ over $p53^{f/f}$.

**AM-pole**	*m/z*	P-value	Average *$p53^{d/d}$*	StDev *$p53^{d/d}$*	Average *$p53^{f/f}$*	StDev *$p53^{f/f}$*	*$p53^{d/d}$ /$p53^{f/f}$*
FA 18:1	281.2465	0.000	0.20	0.012	0.25	0.024	0.80
FA 20:5	301.2152	0.001	0.04	0.004	0.03	0.007	1.36
FA 20:4	303.2308	0.023	0.21	0.026	0.18	0.023	1.15
FA 20:1	309.2777	0.000	0.01	0.001	0.01	0.002	0.64
FA 22:6	327.2304	0.009	0.10	0.004	0.07	0.028	1.37
FA 24:1	365.3402	0.010	0.00	0.001	0.00	0.001	0.61

**AM-pole**	*m/z*	P-value	Average *$p53^{d/d}$*	StDev *$p53^{d/d}$*	Average $p53^{f/f}$	StDev *$p53^{f/f}$*	*$p53^{d/d}$ /$p53^{f/f}$*
PC 36:5	818.516	0.01	0.01	0.002	0.01	0.001	1.27
PC 36:4	820.5332	0.03	0.10	0.01	0.10	0.004	1.05
PC 42:6	900.5972	0.04	0.0003	0.0002	0.0001	0.0001	2.39

**AM-pole**	*m/z*	P-value	Average *$p53^{d/d}$*	StDev *$p53^{d/d}$*	Average *$p53^{f/f}$*	StDev *$p53^{f/f}$*	*$p53^{d/d}$ /$p53^{f/f}$*
MG 16:0	369.2433	0.026	0.06	0.03	0.10	0.05	0.59
MG 18:1	395.2584	0.0004	0.13	0.02	0.16	0.02	0.80
MG 18:0	397.2746	0.008	0.02	0.01	0.05	0.02	0.45
MG 20:4	417.2437	0.001	0.14	0.03	0.10	0.009	1.38
MG 20:3	419.2587	0.014	0.005	0.003	0.008	0.002	0.60
MG 20:2	421.2751	0.007	0.00002	0.0001	0.001	0.0005	0.04
MG 22:6	441.244	0.005	0.17	0.03	0.13	0.02	1.26

**AM-pole**	*m/z*	P-value	Average *$p53^{d/d}$*	StDev *$p53^{d/d}$*	Average *$p53^{f/f}$*	StDev *$p53^{f/f}$*	*$p53^{d/d}$ /$p53^{f/f}$*
DG 32:1	605.4611	0.0001	0.006	0.003	0.01	0.003	0.47
DG 34:1	633.4921	0.0002	0.08	0.02	0.11	0.013	0.75
DG 36:4	655.4767	0.008	0.14	0.03	0.11	0.014	1.29
DG 38:5	681.4925	0.01	0.04	0.009	0.029	0.013	1.46
DG 40:8	703.4782	0.002	0.03	0.02	0.008	0.006	3.94
DG 40:7	705.4967	0.0009	0.03	0.01	0.008	0.006	3.31

M-pole	*m/z*	P-value	Average *p53*$^{d/d}$	StDev *p53*$^{d/d}$	Average *p53*$^{f/f}$	StDev *p53*$^{f/f}$	*p53*$^{d/d}$ /*p53*$^{f/f}$
FA 18:1	281.2465	0.001	0.37	0.034	0.43	0.034	0.85
FA 20:1	309.2777	0.001	0.02	0.002	0.03	0.007	0.70
FA 22:6	327.2304	0.001	0.06	0.007	0.04	0.015	1.60
FA 22:5	329.2462	0.000	0.03	0.003	0.03	0.003	1.31
FA 22:4	331.2619	0.006	0.05	0.006	0.04	0.007	1.26

M-pole	*m/z*	P-value	Average *p53*$^{d/d}$	StDev *p53*$^{d/d}$	Average *p53*$^{f/f}$	StDev *p53*$^{f/f}$	*p53*$^{d/d}$ /*p53*$^{f/f}$
PC 40:8	868.5305	0.04	0.002	0.0005	0.0012	0.0004	1.35
PC 40:6	872.5618	0.05	0.024	0.0050	0.0200	0.0041	1.21
PC 40:4	876.5968	0.03	0.002	0.0003	0.0020	0.0003	1.14
PC 42:7	898.5808	0.02	0.001	0.0001	0.0005	0.0001	1.23
PC 42:6	900.5972	0.00	0.001	0.0001	0.0004	0.0001	1.47

M-pole	*m/z*	P-value	Average *p53*$^{d/d}$	StDev *p53*$^{d/d}$	Average *p53*$^{f/f}$	StDev *p53*$^{f/f}$	*p53*$^{d/d}$ /*p53*$^{f/f}$
MG 16:0	369.2433	0.002	0.09	0.05	0.19	0.08	0.47
MG 20:4	417.2437	0.005	0.17	0.03	0.13	0.04	1.37

M-pole	*m/z*	P-value	Average *p53*$^{d/d}$	StDev *p53*$^{d/d}$	Average *p53*$^{f/f}$	StDev *p53*$^{f/f}$	*p53*$^{d/d}$ /*p53*$^{f/f}$
DG 36:3	657.492	0.03	0.13	0.13	0.09	0.04	1.44
DG 40:5	709.533	0.04	0.01	0.01	0.004	0.01	2.74

1. Hirota Y*, et al.* (2010) Uterine-specific p53 deficiency confers premature uterine senescence and promotes preterm birth in mice. *The Journal of clinical investigation* 120(3):803-815.
2. Patterson NH, Thomas A, & Chaurand P (2014) Monitoring time-dependent degradation of phospholipids in sectioned tissues by MALDI imaging mass spectrometry. *J Mass Spectrom* 49(7):622-627.
3. Lanekoff I*, et al.* (2013) High-speed tandem mass spectrometric in situ imaging by nanospray desorption electrospray ionization mass spectrometry. *Anal Chem* 85(20):9596-9603.
4. Lanekoff I*, et al.* (2012) Automated platform for high-resolution tissue imaging using nanospray desorption electrospray ionization mass spectrometry. *Anal Chem* 84(19):8351-8356.
5. Lanekoff I, Stevens SL, Stenzel-Poore MP, & Laskin J (2014) Matrix effects in biological mass spectrometry imaging: identification and compensation. *Analyst* 139(14):3528-3532.
6. Cha J*, et al.* (2013) Combinatory approaches prevent preterm birth profoundly exacerbated by gene-environment interactions. *The Journal of clinical investigation* 123(9):4063-4075.
